# Computational Study of Catalytic Urethane Formation

**DOI:** 10.3390/polym14010008

**Published:** 2021-12-21

**Authors:** Hadeer Q. Waleed, Marcell Csécsi, Rachid Hadjadj, Ravikumar Thangaraj, Dániel Pecsmány, Michael Owen, Milán Szőri, Zsolt Fejes, Béla Viskolcz, Béla Fiser

**Affiliations:** 1 Institute of Chemistry, University of Miskolc, 3515 Miskolc, Hungary; kemHader@uni-miskolc.hu (H.Q.W.); csecsi.marcell2@gmail.com (M.C.); hrachid.chemeng@gmail.com (R.H.); ravikumar8019@gmail.com (R.T.); pecsmany.daniel@gmail.com (D.P.); michael.owen@uni-miskolc.hu (M.O.); milan.szori@uni-miskolc.hu (M.S.); zsolt.fejes@gmail.com (Z.F.); bela.viskolcz@uni-miskolc.hu (B.V.); 2 Higher Education and Industrial Cooperation Centre, University of Miskolc, 3515 Miskolc, Hungary; 3 Ferenc Rákóczi II, Transcarpathian Hungarian College of Higher Education, 90200 Berehove, Transcarpathia, Ukraine

**Keywords:** catalysts, urethane formation, catalyst-free, DFT, proton affinities, composite method

## Abstract

Polyurethanes (PUs) are widely used in different applications, and thus various synthetic procedures including one or more catalysts are applied to prepare them. For PU foams, the most important catalysts are nitrogen-containing compounds. Therefore, in this work, the catalytic effect of eight different nitrogen-containing catalysts on urethane formation will be examined. The reactions of phenyl isocyanate (PhNCO) and methanol without and in the presence of catalysts have been studied and discussed using the G3MP2BHandHLYP composite method. The solvent effects have also been considered by applying the SMD implicit solvent model. A general urethane formation mechanism has been proposed without and in the presence of the studied catalysts. The proton affinities (PA) were also examined. The barrier height of the reaction significantly decreased (∆*E*_0_ > 100 kJ/mol) in the presence of the studied catalysts, which proves the important effect they have on urethane formation. The achieved results can be applied in catalyst design and development in the near future.

## 1. Introduction

The first fiber-forming polyurethane (PU) [1] was developed by Otto Bayer to compete with Nylon. Bayer’s invention ranks among the most important breakthroughs in polymer science [2]. Polyurethanes are some of the most versatile and unique polymers used in the industry for manufacturing a wide variety of products [3]. Their applications include flexible and rigid foams, paints, coatings, adhesives, packaging, insulation, and synthetic fibers for clothing [4,5,6]. Over two million tons of PU are synthesized every year in the European Union [7]. PUs are formed by reacting di- or polyisocyanates (-N=C=O groups), which are responsible for the rigidity of the polymers, with polyols (-OH groups), which are considered to be the soft segments of the material [8]. Catalysts used in PU synthesis are mainly nitrogen-containing molecules (e.g., amines), inorganic salts, organophosphorus, and organometallic compounds [9]. However, amine catalysts and organometallic compounds are the most widely used in the synthesis of polyurethanes and their raw materials [10]. Previous studies have examined the reaction of methanol with methyl isocyanate at the B3LYP/6-311++G(df,p) level of theory and found that the activity of the reacting system increases with the degree of methanol association [11]. Moreover, another study examined urethane formation (methyl isocyanate and methanol) in the presence of a tertiary amine, 1,4-diazabicyclo[2.2.2]octane (DABCO), as a catalyst [12]. By using DFT calculations, it was found that catalytic urethane formation can be described as an accelerated direct addition [12]. Furthermore, it was determined that 2,4-toluene diisocyanate is slightly more active than methyl isocyanate [13]. Moreover, another combined computational and experimental study suggests that urethane formation can occur through self-catalysis as well [14]. Although several experimental and computational studies have been carried out to understand urethane formation, there is still room for deeper understanding, which is inevitable for catalyst design and development. Therefore, in this paper, catalytic urethane formation will be examined, and the activity of eight different commonly used nitrogen-containing catalysts will be compared by applying density functional theory (DFT) and composite methods. Furthermore, the catalyst-free system will also be studied and used as a reference during the analysis. Thus, the catalytic urethane formation will be described at the molecular level with the aim of supporting future catalyst design and development.

## 2. Methods

Preliminary calculations on the studied system have been carried out using different density functional theory (DFT) methods including B3LYP, BHandHLYP, and wB97X-D [15,16,17] in combination with the 6-31G(d) basis set in gas phase using the Gaussian 09 program package [18]. Furthermore, the effects of two different solvents, tetrahydrofuran (THF, ε_r_ = 7.4257) and acetonitrile (MeCN, ε_r_ = 35.688), have also been considered using the SMD solvent model. Frequency calculations were also carried out to determine the thermodynamic properties of the studied species. All the critical points on the potential energy surfaces (PES) of the studied catalytic processes were found only with the BHandHLYP/6-31G(d) level of theory. Thus, to further improve the accuracy of the results, the G3MP2BHandHLYP composite method [19,20,21] was applied and used in the discussion of the results. To achieve G3MP2BHandHLYP energies, geometry optimization and frequency calculation have been carried out at the BHandHLYP/6-31G(d) level of theory. Furthermore, on the optimized structures, two single-point energy calculations have been performed at the QCISD(T)/6-31G(d) and MP2/GTMP2Large levels of theory. Thus, the G3MP2BHandHLYP total zero-point energy (*E*_0_) has been computed as follows [19,20,21,22]:

*E*_0_[G3MP2BHandHLYP] = *E*[QCISD(T)/6-31G(d)] + *E*[MP2/GTMP2Large]

−*E*[MP2/6-31G(d)] + ∆*E*(HLC) + ∆*E*(ZPE) (1)

where ZPE is the zero-point correction, while HLC is the higher-level correction. HLC is a linear function of the number of valence electrons with α and β spins, −An_β_ − B (n_α_−n_β_), with n_α_ ≥ n_β_. The value of A is 10.041 mE_h_, and B is 4.995 mE_h_ (E_h_−Hartree) for molecules. The catalytic reaction mechanisms have been analyzed and the corresponding structures (minima, intermediates, and transition states) have been located [23]. Intrinsic reaction coordinate (IRC) calculations were also performed in both forward and reverse directions to verify that the transition states (TS) connect the right minima of the proposed potential energy surface [24]. Furthermore, the proton affinities (PAs) of the catalysts were calculated as follows [25]:

PA = −∆*H*_r_ = −(*H* (protonated molecule) − *H* (neutral molecule)) (2)

## 3. Results and Discussion

**Figure 1:**
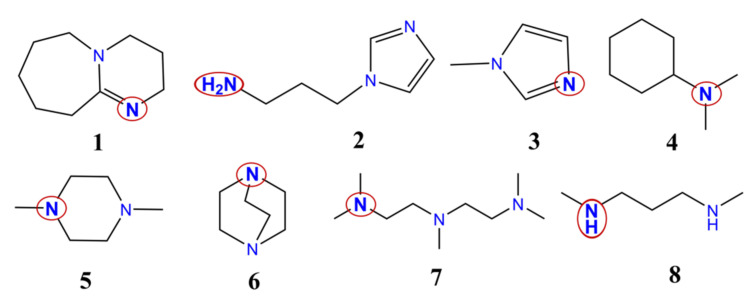
Chemical structures of the studied catalysts. **1**—1,8-diazabicyclo[5,4,0]undec-7-ene; **2**—1-(3-aminopropyl)imidazole; **3**—1-methylimidazole; **4**—*N,N*-dimethylcyclohexanamine; **5**—1,4-dimethylpiperazine; **6**—1,4-diazabicyclo[2.2.2]octane; **7**—*N,N,N′,N″,N″–*pentamethyldiethylenetriamine; **8**—*N,N*-dimethyl-1,3-propanediamine. The catalytic nitrogen-containing groups which are considered in the calculations are highlighted with red circles.

All in all, the catalytic activities of eight different catalysts used in polyurethane synthesis have been compared using theoretical methods. The catalysts contain different amine, ketimine, and aromatic nitrogen functional groups. They are usually applied to synthesize polyurethanes with various mechanical properties (rigid and flexible foams, etc.) (Figure 1).

To determine the effect of the catalysts, a catalyst-free reaction has also been investigated.

### 3.1. Catalyst-Free Reaction Mechanism of Urethane Formation

**Scheme 1:**
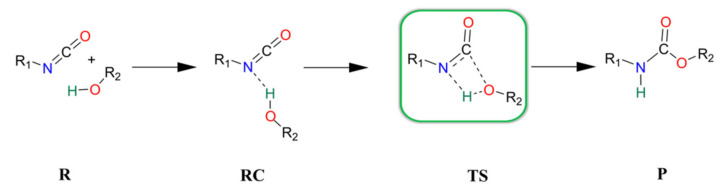
Schematic representation of the general catalyst-free reaction of isocyanates and alcohols. R—reactants, RC—reactant complex, TS—transition state, and P—product.

The catalyst-free urethane bond formation goes through a concerted mechanism. First, a reactant complex (RC) is formed between the isocyanate and alcohol, where the former is an electrophile, and the latter is a nucleophile (Scheme 1). In the transition state (TS), the N=C=O group bends, activating the carbon for the formation of a new C-O bond, while the H-O bond breaks and the N-H forms to achieve the product (P) with the urethane bond.

### 3.2. Structural and Energetic Features of a Catalyst-Free Model Reaction

**Figure 2:**
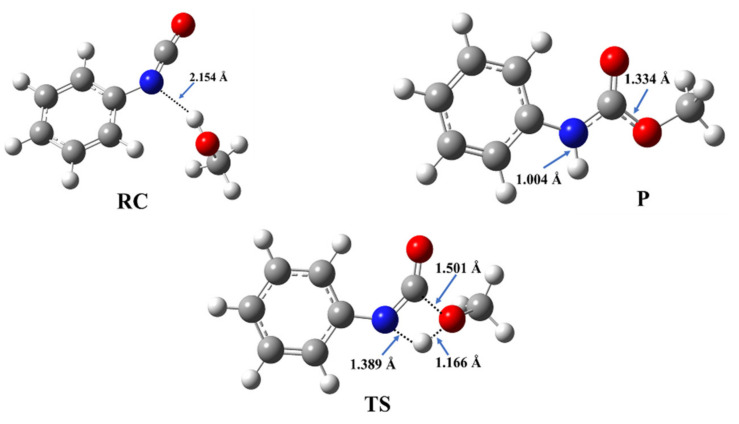
Three-dimensional structures of the reactant complex (RC), transition state (TS), and product (P) in the reaction of methanol and phenyl isocyanate, which are used as a model of catalyst-free urethane formation. The structures have been optimized at the BHandHLYP/6-31G(d) level of theory in acetonitrile at 298.15 K and 1 atm.

The reaction of methanol and phenyl isocyanate has been selected as a model to describe the energetic and structural features of the catalyst-free urethane formation. The structures of the corresponding reactant complex (RC), transition state (TS), and product (P) have been optimized (Figure 2) and the reaction mechanism has been characterized.

**Figure 3:**
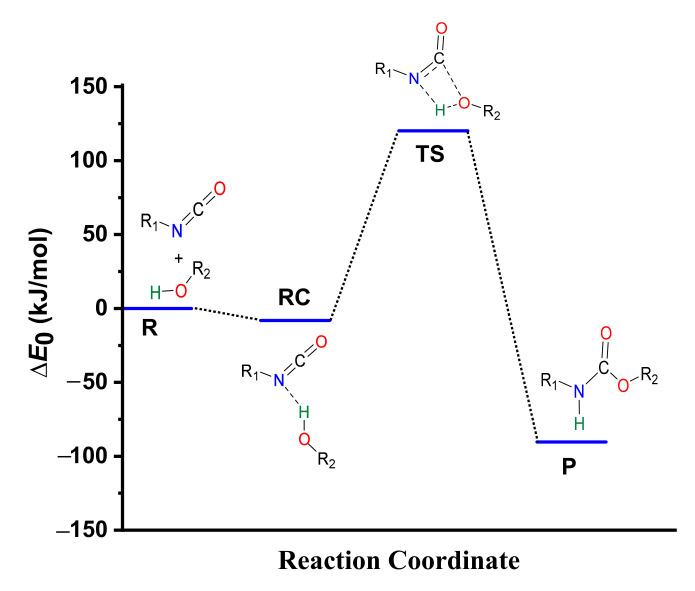
Energy profile (zero-point corrected) of the phenyl isocyanate and methanol reaction calculated using the G3MP2BHandHLYP composite method in acetonitrile, using the SMD implicit solvent model at 298.15 K and 1 atm.

During the reaction between phenyl isocyanate and methanol, a reactant complex (RC) forms in the first step. It is stabilized by a hydrogen bond between the hydroxyl group of the methanol and the nitrogen of the isocyanate group with a corresponding distance of 2.154 Å (Figure 2), while the zero-point corrected relative energy is −8.22 kJ/mol compared to the sum of the separated reactants (Table 1, Tables S2 and S3, and Figure 3).

After this, in the TS, a proton from the hydroxyl group shifts to the nitrogen atom of the isocyanate, while a bond forms between the hydroxyl’s oxygen and the NCO’s carbon. The potential energy curve of the reaction (Figure 3) shows that the reaction needs to overcome a barrier of 120.18 kJ/mol to reach the final urethane product (P), which has a relative energy of −90.33 kJ/mol (Table 1).

### 3.3. Urethane Formation Reaction in the Presence of Nitrogen-Containing Catalysts 

A varied set of nitrogen-containing catalysts have been selected from the literature [9,26,27,28,29,30,31]. Based on their applications, the catalysts can be divided into four groups as follows: 1, 2–4, 5–7, and 8, which are used in the synthesis of flexible, rigid, and semi-rigid foams, and others, respectively (Figure 1 and Table 2).

There are three linear, three cyclic, one aromatic, and one aromatic and linear structures. Catalyst 4 has only one, while all the others have at least two potential catalytic sites (Table 2). Most of the catalysts have at least one tertiary amine group, except 8, which contains only two secondary amines (Figure 1). Secondary ketimine groups are also important, as occurs in catalyst 1, while catalysts 2 and 3 contain aromatic groups. Primary and secondary amine groups can be found only in catalysts 2 and 8, and they are significant due to their potential reaction with isocyanates. However, only the catalytic activity of the species will be discussed.

### 3.4. Urethane Formation Mechanism in the Presence of Catalysts

**Scheme 2:**
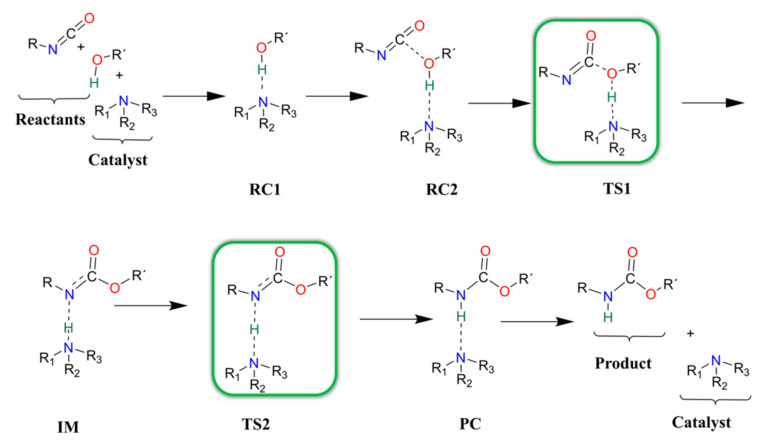
Schematic representation of the proposed general reaction mechanism of isocyanates and alcohols in the presence of nitrogen-containing catalysts.

In each catalyst, only one catalytic site is considered during the computational study of the catalytic reaction. Previous studies which described catalytic urethane formation using DFT calculations were used as a starting point [12,13], but changes had to be implemented in the choice of method as not all of the suggested levels of theory were able of locating the correct minima and transition states (e.g., proton shift) on the potential energy surface of the reaction. After an extensive method test (see Methods), it was found that only BHandHLYP/6-31G(d) in combination with acetonitrile solvent, mimicked by the SMD solvent model, was able to locate the correct structures. To further improve the accuracy of this level of theory, the G3MP2BHandHLYP protocol was applied, and the corresponding results are discussed. The proposed and studied catalytic urethane formation reaction mechanism contains seven steps (Scheme 2).

It starts with the formation of a complex between the alcohol and the catalyst (RC1) because, under industrial conditions, the catalyst is first mixed into the polyols. In the next step, the isocyanate is mixed into the system and a three-molecule complex is formed (RC2). After the complex formation, a proton transfer occurs between the alcohol and the amine group of the catalyst (TS1). At the same time, a new C-O bond forms between the carbon of the NCO group and the oxygen of the alcohol, which leads to the formation of an intermediate (IM). Thereafter, the catalyst will return the proton through a transition state (TS2) and, thus, a product complex is formed (PC), where the urethane bond is complete and the catalyst is hydrogen-bonded to the product. In the final step, the catalysts and the product will be separated (P).

### 3.5. Proton Affinity (PA) of the Studied Catalysts

As the proposed catalytic mechanism contains protonation steps, it is important to know the proton affinities (PA) of the catalysts. Only the nitrogen-containing groups of the catalysts are considered, and the corresponding PA values have been calculated (Table 3 and Table S1).

In some cases, there are more than one amine group in the catalysts, but only one of them is considered during the catalytic mechanism. However, the proton affinity has been computed for all nitrogen-containing groups. The experimental and computational results are in fairly good agreement with each other (Table 3 and Table S1). In the case of catalyst 4, the deviation between the calculated and experimental values was the lowest, only 15.08 kJ/mol. The deviation between the calculated and literature data is the highest, 68.66 kJ/mol, in the case of catalyst 8. Catalyst 3 has the lowest calculated proton affinity (785.51 kJ/mol) within the studied set of structures in the case of its tertiary amine group (Table 3 and Table S1), which makes it the best proton donor, as less energy is needed to remove the proton. It is followed by the tertiary amine group of catalyst 2, which has just a slightly higher PA value (800.93 kJ/mol). The highest proton affinity belongs to the secondary ketimine of catalyst 1 (1070.34 kJ/mol), which makes it the best proton acceptor. Most of the calculated PA values are around or above 900 kJ/mol (Table 3 and Table S1). All in all, the proton affinities of the amine groups of the catalysts cover a wide range of almost 300 kJ/mol, between 785.5 and 1070.3 kJ/mol.

### 3.6. Structural Features of Urethane Formation in the Presence of the Studied Catalysts

Even though the same reaction mechanism of phenyl isocyanate and methanol was considered in the presence of the studied catalysts (Scheme 2), the structural features vary slightly with the different catalysts (Table 4, Figure 4 and Figures S1–S7).

**Figure 4:**
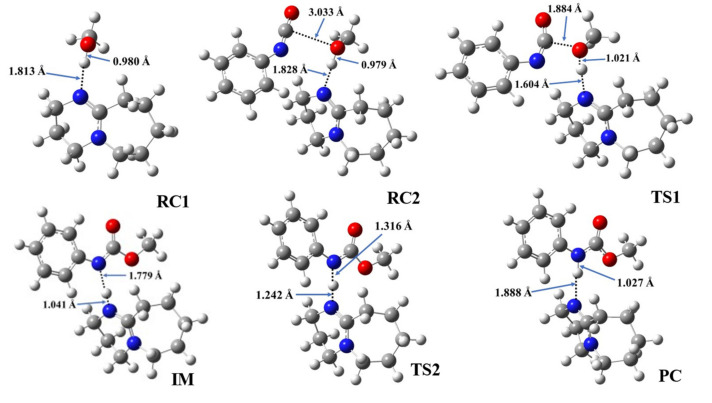
Optimized structures along the reaction pathway between phenyl isocyanate and methanol in the presence of catalyst 1, calculated at the BHandHLYP/6-31G(d) level of theory in acetonitrile at 298.15 K and 1 atm. RC—reactant complex, TS—transition state, IM—intermediate, and PC—product complex.

In the first step, during which the RC1 forms, the distance between the catalyst’s nitrogen and the methanol’s hydroxyl hydrogen ranged between 1.812 and 1.882 Å (Table 4, N-H*). In the second step, RC2, a three-molecule complex forms with the addition of the PhNCO. A new interaction between the methanol’s oxygen and the PhNCO’s isocyanate group occurs, but only minor changes could be identified in the length of the previously established N-H*. The effect on the O-H bond length was even smaller and almost no change was observed between RC1 and RC2 (Table 4). As the TS1 developed in the presence of the catalysts, the N-H slightly decreased while the C-O significantly decreased, by ~0.2 and ~1.2 Å, respectively. At the same time, the O-H increased in each case by ~0.3 Å (Figure 4 and Figures S1–S7). The structural changes take place according to the proposed steps, and it shows that the methanol’s oxygen loses the proton while attaching itself to the isocyanate’s N=C=O group. Thus, an IM is formed including the protonated catalyst (N-H*), which is hydrogen-bonded to the nitrogen of the adduct (N-H**), while the methoxy group (from the methanol) is attached to the carbon of the former isocyanate group (C-O) (Figure 4 and Figures S1–S7). The N-H* distance was the lowest in this step for each catalytic process, and it was in the range of 1.041 and 1.092 Å, while N-H** ranged between 1.597 and 1.779 Å. In TS2, proton transfer occurs and, thus, the final urethane bond develops (N-H* increases, while N-H** decreases). Through this step, the PC will be formed, which is a bimolecular complex of the catalyst and the final product (methyl phenylcarbamate).

### 3.7. Energetics of Urethane Formation in the Presence of Different Catalysts

**Figure 5:**
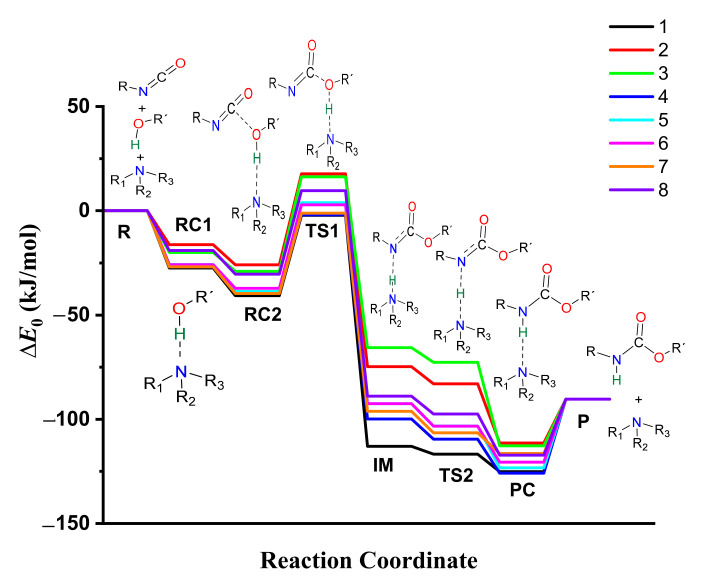
Energy profiles (zero-point corrected, ∆*E*_0_) of the catalyzed urethane formation reactions calculated with the G3MP2BHandHLYP composite method in acetonitrile, using the SMD implicit solvent model at 298.15 K and 1 atm.

The thermodynamic properties of the catalyzed isocyanate–methanol reaction have been computed (Table 1, Tables S2 and S3) and the corresponding energy profiles have been drawn (Figure 5 and Figure S10). The most stable methanol-catalyst complex (RC1, ∆*E*_0_ = −27.43 kJ/mol) and trimolecular complex (RC2, ∆*E*_0_ = −40.71 kJ/mol) were formed in the presence of catalyst 1, while the least stable ones were associated with catalyst 2 (RC1 and RC2, ∆*E*_0_ = −16.27 and −25.95 kJ/mol, respectively). The calculated barrier height significantly decreased (∆∆*E*_0_ > 100 kJ/mol) in the presence of the studied set of catalysts compared to the catalyst-free reaction. The relative energy at TS1 was the lowest in case of catalyst 1 (∆*E*_0_ = −2.20 kJ/mol) within the studied set of catalysts (Table 1, Figure 5, and Figure S9).

The relative energy of the IM was the highest in the presence of catalyst 3 (∆*E*_0_ = −65.36 kJ/mol), while this step was more preferred in the case of catalyst 1. By applying catalyst 1, TS2 (proton shift) had the lowest relative energy (∆*E*_0_ = −116.72 kJ/mol) compared to the studied set of catalysts. Before the reaction completed, the product complex (PC) formed with a relative energy range of −111.38 and −125.82 kJ/mol. After this, the product was achieved, which had a relative energy of −90.33 kJ/mol. Therefore, along with the whole reaction mechanism, catalyst 1 was the most effective and provided the most favorable pathway, which can be associated with its high PA and cyclic structure.

## 4. Conclusions

Urethane formation in catalyst-free and catalytic processes was studied using computational chemical tools, and a general formation mechanism was proposed in the presence of eight nitrogen-containing catalysts. An extensive method test was carried out and BHandHLYP/6-31G(d) was selected in combination with the SMD solvent model to describe all the different steps along the pathways of the catalytic urethane formation mechanism. Further improvements on the calculations were included using the G3MP2BHandHLYP composite calculation scheme. The phenyl isocyanate–methanol reaction was used as a model system for the calculations. The catalytic activities of eight different catalysts used in polyurethane synthesis have been compared by adding them to the model system. As the studied catalytic mechanism contains protonation steps, the proton affinities (PA) of the catalysts have also been calculated and compared. All in all, the proton affinities of the amine groups of the catalysts cover a wide range of almost 300 kJ/mol, between 760.6 and 1070.34 kJ/mol. Structural changes along the reaction coordinates have also been discussed and compared. The effects of primary, secondary, and tertiary amine groups have been analyzed. The energetics of the model reaction significantly changed in the presence of catalysts. The barrier height reduced by >100 kJ/mol. These results show that catalysts are key components in polyurethane synthesis, and significantly reduce the environmental impact of chemical processes and the energy required to carry them out. It was found that the most effective catalyst is a cyclic secondary ketimine (catalyst 1), and the proton affinity of the species plays an important role in its catalytic properties. These findings can be applied in catalyst design and development.

## Figures and Tables

**Table 1:** Zero-point corrected relative energies (∆*E*_0_) of the reaction between phenyl isocyanate and methanol with and without catalysts, calculated using the G3MP2BHandHLYP composite method in acetonitrile, using the SMD implicit solvent model at 298.15 K and 1 atm. Cat. —catalyst, R—reactant, RC—reactant complex, TS—transition state, IM—intermediate, PC—product complex, and P—product.

	∆*E*_0_ (kJ/mol)							
	R	RC1	RC2	TS1	IM	TS2	PC	P
**Cat.-free**	0.00	-	−8.22 *	120.18	-	-	-	−90.33
**1**	0.00	−27.43	−40.71	−2.20	−112.98	−116.72	−125.14	−90.33
**2**	0.00	−16.27	−25.95	17.72	−74.79	−82.97	−111.38	−90.33
**3**	0.00	−19.99	−29.04	16.33	−65.63	−72.68	−112.74	−90.33
**4**	0.00	−26.81	−38.76	−1.96	−99.90	−109.55	−125.82	−90.33
**5**	0.00	−25.77	−38.73	3.84	−92.55	−103.27	−123.31	−90.33
**6**	0.00	−25.85	−37.16	2.85	−92.53	−103.34	−120.54	−90.33
**7**	0.00	−26.88	−39.61	−1.08	−96.15	−106.49	−116.42	−90.33
**8**	0.00	−19.03	−30.45	9.63	−88.89	−97.52	−117.25	−90.33

* RC for catalyst-free reaction.

**Table 2:** Structural features of the studied catalysts and their applications. Cat.—catalyst, RNH_2_, R_2_NH, R_3_N, R_2_C=N-R: primary, secondary, tertiary amines, and secondary ketimine, respectively.

Cat.	Resulted Polyurethane Type	Structural Property of PU	Functional Groups				References
			RNH_2_	R_2_NH	R_3_N	R_2_C=N-R	
**1**	Flexible foam	Cyclic			•	•	[9]
**2**	Rigid foam	Aromatic, Linear	•	•	•		[9]
**3**	Rigid foam	Aromatic		•	•		[26]
**4**	Rigid foam	Linear			•		[27]
**5**	Semi-rigid	Cyclic			• •		[28,29]
**6**	Semi-rigid	Cyclic			• •		[30]
**7**	Semi-rigid	Linear			• • •		[9]
**8**	Other	Linear		• •			[31]

**Table 3:** Computed (PA_calc_) and measured proton affinities (PAexp) in kJ/mol. The calculations have been carried out at the G3MP2BHandHLYP level of theory in gas phase at 298.15 K and 1 atm. RNH_2_, R_2_NH, R_3_N, R_2_C=N-R: primary, secondary, tertiary amines, secondary ketimine, respectively. • side; •• middle amine group.

Catalysts	PA_calc_ (kJ/mol)				PA_exp_ (kJ/mol) [32]
	RNH_2_	R_2_NH	R_3_N	R_2_C=N-R	
**1**	-	-	937.50	1070.34	-
**2**	905.75	994.19	800.93	-	-
**3**	-	983.68	785.51	-	959.6
**4**	-	-	998.68	-	983.6
**5**	-	-	984.84	-	-
**6**	-	-	983.91	-	963.4
**7**	-	-	984.71 • 982.62 ••	-	-
**8**	-	966.54	-	-	1035.2

**Table 4:** The N-H, O-H, and C-O bond lengths (Å) along the phenyl isocyanate (PhNCO) and methanol reaction pathway in the presence of the studied catalysts. N-H* for catalysts, while N-H** for PhNCO.

Cat.	RC1		RC2			TS1		IM		TS2			PC	
	N-H*	O-H	N-H*	O-H	C-O	N-H*	O-H	C-O	N-H*	N-H**	N-H*	N-H**	N-H*	N-H**
**1**	1.813	0.980	1.828	0.979	3.033	1.604	1.021	1.884	1.041	1.779	1.242	1.316	1.888	1.027
**2**	1.848	0.977	1.853	0.977	3.026	1.653	1.011	1.869	1.092	1.605	1.168	1.437	1.920	1.026
**3**	1.882	0.972	1.855	0.974	3.064	1.690	1.002	1.828	1.088	1.597	1.134	1.481	1.967	1.019
**4**	1.864	0.977	1.868	0.977	3.036	1.670	1.014	1.845	1.065	1.725	1.223	1.370	2.012	1.023
**5**	1.879	0.975	1.883	0.975	3.062	1.695	1.009	1.836	1.068	1.718	1.212	1.384	2.022	1.021
**6**	1.851	0.976	1.827	0.980	3.078	1.657	1.013	1.857	1.073	1.670	1.211	1.372	1.996	1.023
**7**	1.870	0.976	1.872	0.976	3.053	1.669	1.012	1.845	1.070	1.703	1.213	1.381	1.987	1.023
**8**	1.849	0.978	1.850	0.978	3.052	1.653	1.013	1.866	1.077	1.660	1.203	1.387	1.927	1.026

## Data Availability

The structures, figures, and additional tables are available in the supporting information.

## Supplementary Materials

The following are available online at https://www.mdpi.com/article/10.3390/polym14010008/s1, Figures S1–S7: Optimized structures of the catalysts, Figures S8 and S9: Energy profiles of the catalyst-free and catalyzed urethane formations. Table S1: Computed and measured proton affinities (PA), Tables S2 and S3: Zero-point corrected relative energies (∆*E*_0_), relative enthalpies (∆*H*), and relative Gibbs free energies (∆*G*), Table S4: Cartesian coordinates of the stationary points for the reaction system studied.

## References

[1] Szycher M. (2012). Structure–Property Relations in Polyurethanes. Szycher’s Handbook of Polyurethanes.

[2] Szycher M. (1938). Handbook of Polyurethanes.

[3] Cavaco L.I., Melo J.A. (2012). Polyurethanes Properies, Structure and Applicatiovs.

[4] Sabrina S.S.A., Denilson A.S., Danielle M.A. (2008). Physico-Chemical Analysis of Flexible Polyurethane Foams Containing Commercial Calcium Carbonate. Mater. Res..

[5] Akindoyo J.O., Beg M.D.H., Ghazali S., Islam M.R., Jeyaratnam N., Yuvaraj A.R. (2016). Polyurethane Types, Synthesis and Applications—A Review. RSC Adv..

[6] Li S., Xu C., Yang W., Tang Q. (2017). Thermoplastic Polyurethanes Stemming from Castor Oil: Green Synthesis and Their Application in Wood Bonding. Coatings.

[7] Marrs K.D. Bio-Based Polyurethanes-A Computational and Experimental Study. Proceedings of the 8th Visegrad Symposium on Structural Systems Biology.

[8] Gama N.V., Ferreira A., Barros-Timmons A. (2018). Polyurethane Foams: Past, Present, and Future. Materials.

[9] Silva A.L., Bordado J.C. (2004). Recent Developments in Polyurethane Catalysis: Catalytic Mechanisms Review. Catal. Rev. Sci. Eng..

[10] Li R., Liu L., Liu Y., Wang B., Yang J.J., Zhang J. (2018). Research Progress of Amine Catalysts for Polyurethane. Top. Chem. Mater. Eng..

[11] Samuilov A.Y., Balabanova F.B., Kamalov T.A., Samuilov Y.D., Konovalov A.I. (2010). Quantum-Chemical Study on Reactions of Isocyanates with Linear Methanol Associates: III.* Reaction of Merhyl Isocyanate with Linear Methanol Associates. Russ. J. Org. Chem..

[12] Hatanaka M. (2011). DFT Analysis of Catalytic Urethanation. Bull. Chem. Soc. Jpn..

[13] Wen Z. (2014). DFT Study of the Catalytic Mechanism for Urethane Formation in the Presence of Basic Catalyst 1,4-Diazabicyclo[2.2.2]Octane. Commun. Comput. Chem..

[14] Cheikh W., Rózsa Z.B., López C.O.C., Mizsey P., Viskolcz B., Szori M., Fejes Z. (2019). Urethane Formation with an Excess of Isocyanate or Alcohol: Experimental and Ab Initio Study. Polymers.

[15] Becke A.D. (1993). Density-Functional Thermochemistry. III. The Role of Exact Exchange. J. Chem. Phys..

[16] Chai J.D., Head-Gordon M. (2008). Long-Range Corrected Hybrid Density Functionals with Damped Atom-Atom Dispersion Corrections. Phys. Chem. Chem. Phys..

[17] Becke A.D. (1993). A New Mixing of Hartree-Fock and Local Density-Functional Theories. J. Chem. Phys..

[18] Frisch M.J., Trucks G.W., Schlegel H.B., Scuseria G.E., Robb M.A., Cheeseman J.R., Scalmani G., Barone V., Mennucci B., Petersson G.A. (2009). Gaussian 09, Revision, E.01.

[19] Szori M., Abou-Abdo T., Fittschen C., Csizmadia I.G., Viskolcz B. (2007). Allylic Hydrogen Abstraction II. H-Abstraction from 1,4 Type Polyalkenes as a Model for Free Radical Trapping by Polyunsaturated Fatty Acids (PUFAs). Phys. Chem. Chem. Phys..

[20] Szori M., Fittschen C., Csizmadia I.G., Viskolcz B. (2006). Allylic H-Abstraction Mechanism: The Potential Energy Surface of the Reaction of Propene with OH Radical. J. Chem. Theory Comput..

[21] Izsák R., Szori M., Knowles P.J., Viskolcz B. (2009). High Accuracy Ab Initio Calculations on Reactions of OH with 1-Alkenes. The Case of Propene. J. Chem. Theory Comput..

[22] Janoschek R., Rossi M.J. (2002). Thermochemical Properties of Free Radicals from G3MP2B3 Calculations. Int. J. Chem. Kinet..

[23] Hadjadj R., Csizmadia I.G., Mizsey P., Jensen S., Viskolcz B., Fiser B. (2020). Water Enhanced Mechanism for CO_2_—Methanol Conversion. Chem. Phys. Lett..

[24] Wang X., HU W., Gui D., Chi X., Wang M., Tian D., Liu J., Ma X., Pang A. (2013). DFT Study of the Proton Transfer in the Urethane Formation between 2,4-Diisocyanatotoluene and Methanol. Bull. Chem. Soc. Jpn. Vol..

[25] Kolboe S. (2014). Proton Affinity Calculations with High Level Methods. J. Chem. Theory Comput..

[26] Beran R., Zarybnicka L., Machova D. (2020). Recycling of Rigid Polyurethane Foam: Micro-Milled Powder Used as Active Filler in Polyurethane Adhesives. J. Appl. Polym. Sci..

[27] Chuayjuljit S., Maungchareon A., Saravari O. (2010). Preparation and Properties of Palm Oil-Based Rigid Polyurethane Nanocomposite Foams. J. Reinf. Plast. Compos..

[28] Samarappuli I.P., Liyanage N.M.V.K. Evaluation of the Suitability of 1,4-Dimethylpiperazine as a Substitute Catalyst in Polyurethane Foam Production. Proceedings of the MERCon 2018—4th International Multidisciplinary Moratuwa Engineering Research Conference.

[29] Kennedy W.A., Austin T. (1972). Catalyst Combination for Polyurethanes. U.S. Patent.

[30] El Ghobary H., Muller L. (2000). Process for Preparing Polyurethane Foam. EP Patent.

[31] Shokuhi A., Ardjmand M. (2008). Studying on the Mechanism and Raw Materials Used to Manufacturing Polyurethane. Chem. Technol. Indian J..

[32] NIST Chemistry WebBook. https://webbook.nist.gov/chemistry/.

