# Supplementary material for: Computational Study of Catalytic Urethane Formation
Source: Polymers (Basel). 2021 Dec 21;14(1):8. doi: 10.3390/polym14010008 (PMC8747140; doi:10.3390/polym14010008)
Supplement: Supplementary file 1 [file polymers-14-00008-s001.zip › polymers-1509272-supplementary.pdf]

polymers

Computational Study of Catalytic Urethane Formation

Hadeer Q. Waleed [1], Marcell Csécsi [1], Rachid Hadjadj [1], Ravikumar Thangaraj [1], Dániel Pecsmány[1,2], Michael Owen [1,2], Milán Szőri [1], Zsolt Fejes [1], Béla Viskolcz [1] and Béla Fiser [1,2,3,*]

[1] Institute of Chemistry, University of Miskolc, 3515 Miskolc-Egyetemváros, Hungary; kemHader@uni-miskolc.hu (H.Q.W.); csecsi.marcell2@gmail.com (M.C.); hrachid.chemeng@gmail.com (R.H.); ravikumar8019@gmail.com (R.T.); pecsmany.daniel@gmail.com (D.P.); michael.owen@uni-miskolc.hu (M.O.); milan.szori@uni-miskolc.hu (M.S.); zsolt.fejes@gmail.com (Z.F.); bela.viskolcz@uni-miskolc.hu (B.V.)
[2] Higher Education and Industrial Cooperation Centre, University of Miskolc, 3515 Miskolc-Egyetemváros, Hungary
[3] Ferenc Rákóczi II, Transcarpathian Hungarian College of Higher Education, 90200 Beregszász, Transcarpathia, Ukraine
* Correspondence: kemfiser@uni-miskolc.hu

Supporting Information

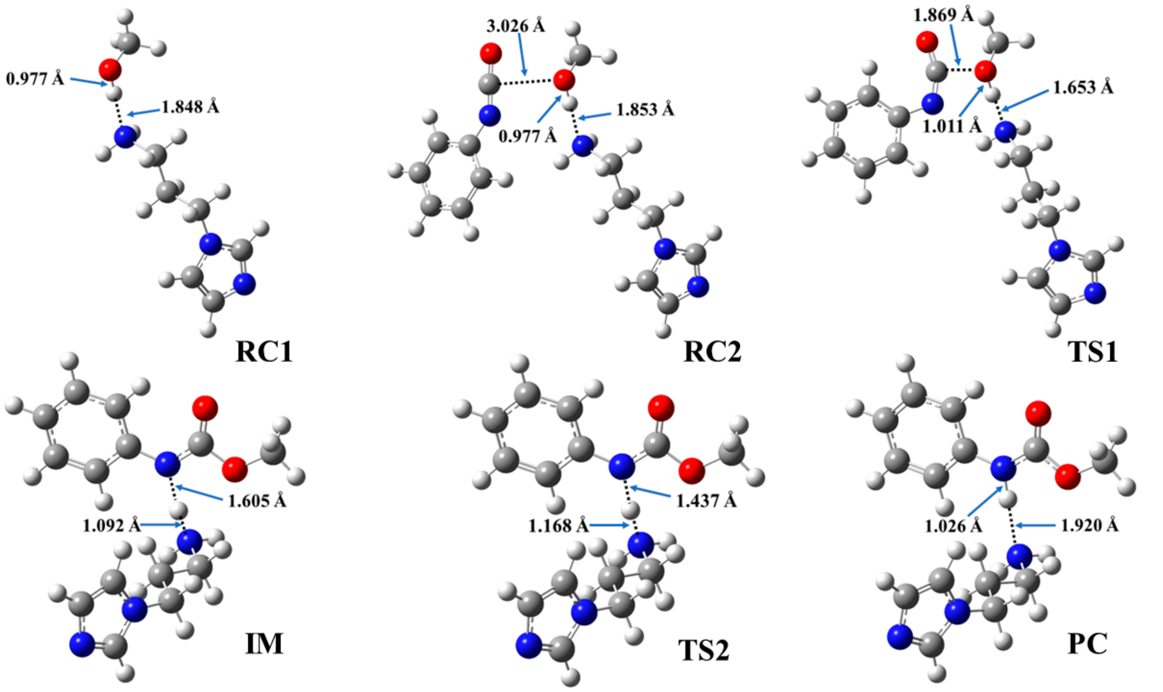

Figure S1. Optimized structures along the reaction pathway between phenyl isocyanate (PhNCO) and methanol in the presence of catalyst **2** calculated at the BHandHLYP/6-31G(d) level of theory in acetonitrile at 298.15 K and 1 atm. RC – reactant complex, TS – transition state, IM – intermediate, PC – product complex.

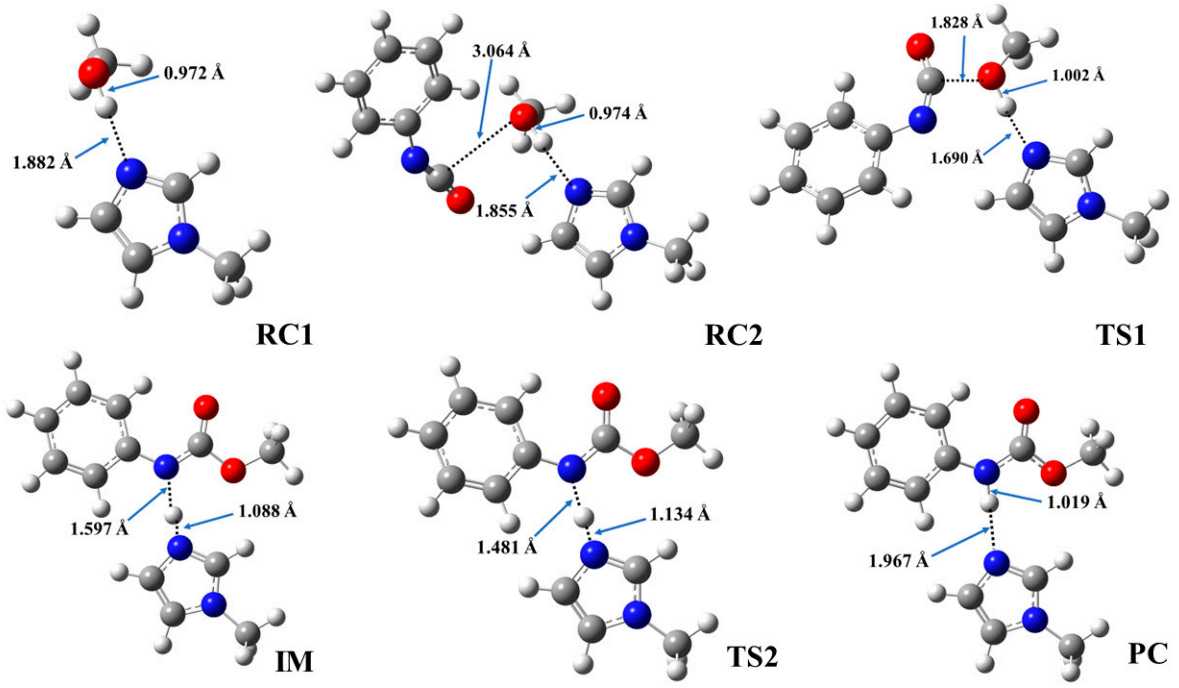

Figure S2. Optimized structures along the reaction pathway between phenyl isocyanate (PhNCO) and methanol in the presence of catalyst **3** calculated at the BHandHLYP/6-31G(d) level of theory in acetonitrile at 298.15 K and 1 atm. RC – reactant complex, TS – transition state, IM – intermediate, PC – product complex.

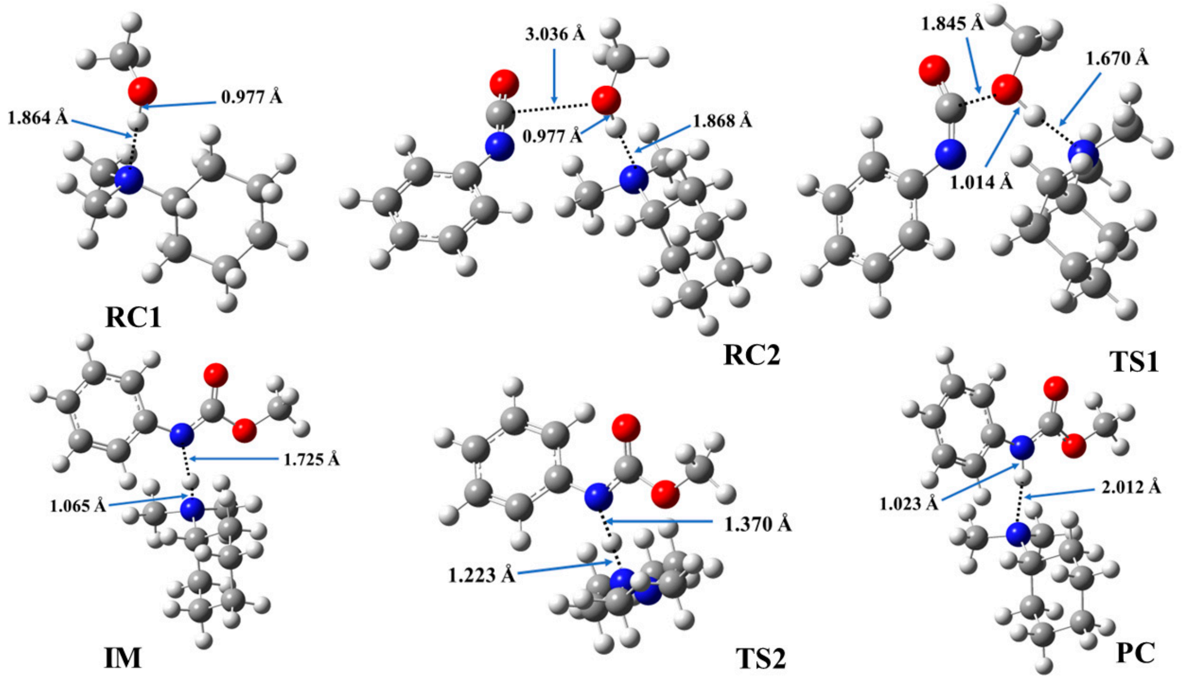

Figure S3. Optimized structures along the reaction pathway between phenyl isocyanate (PhNCO) and methanol in the presence of catalyst **4** calculated at the BHandHLYP/6-31G(d) level of theory in acetonitrile at 298.15 K and 1 atm. RC – reactant complex, TS – transition state, IM – intermediate, PC – product complex.

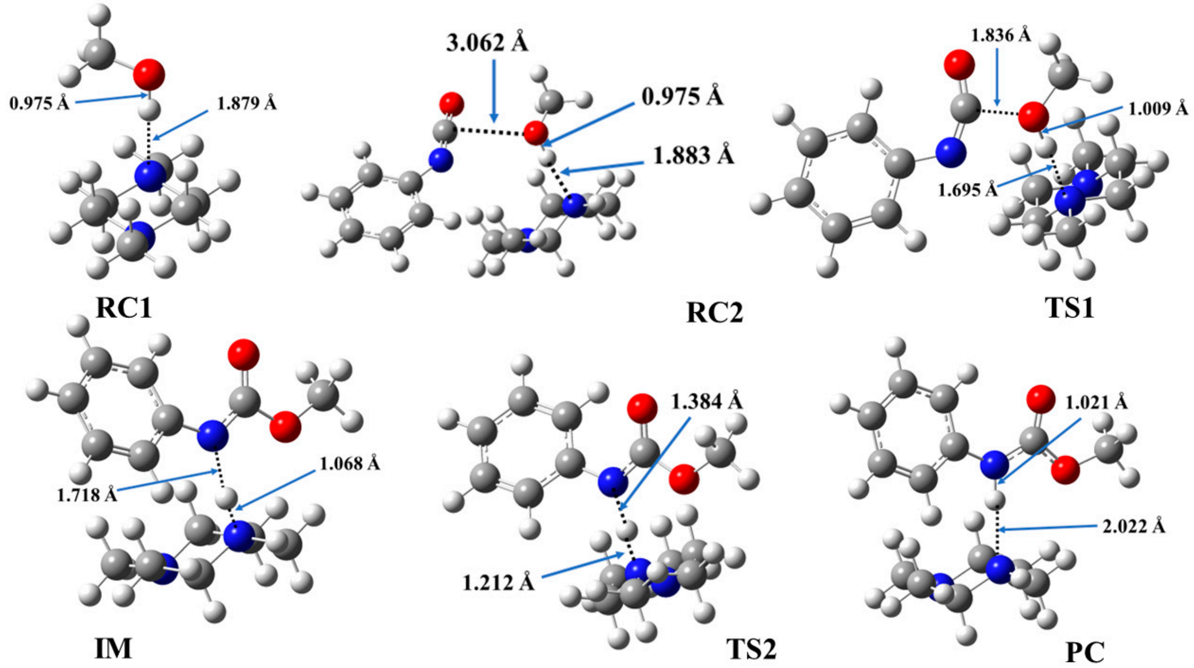

Figure S4. Optimized structures along the reaction pathway between phenyl isocyanate (PhNCO) and methanol in the presence of catalyst **5** calculated at the BHandHLYP/6-31G(d) level of theory in acetonitrile at 298.15 K and 1 atm. RC – reactant complex, TS – transition state, IM – intermediate, PC – product complex.

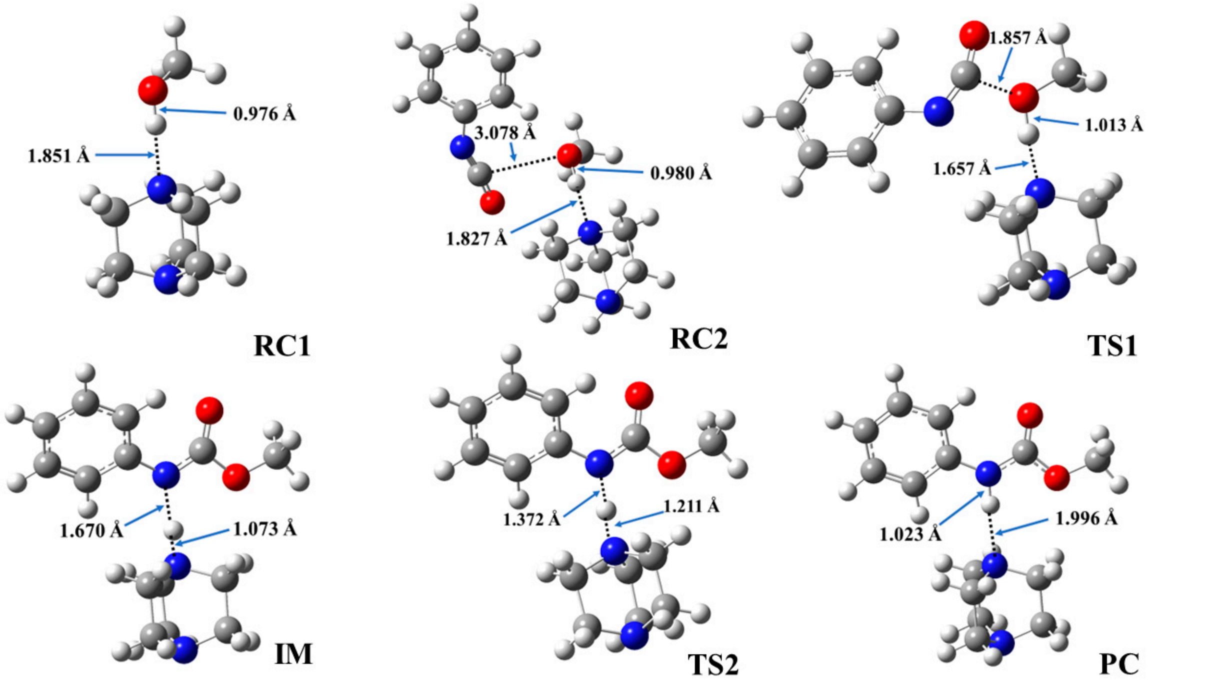

Figure S5. Optimized structures along the reaction pathway between phenyl isocyanate (PhNCO) and methanol in the presence of catalyst **6** calculated at the BHandHLYP/6-31G(d) level of theory in acetonitrile at 298.15 K and 1 atm. RC – reactant complex, TS – transition state, IM – intermediate, PC – product complex.

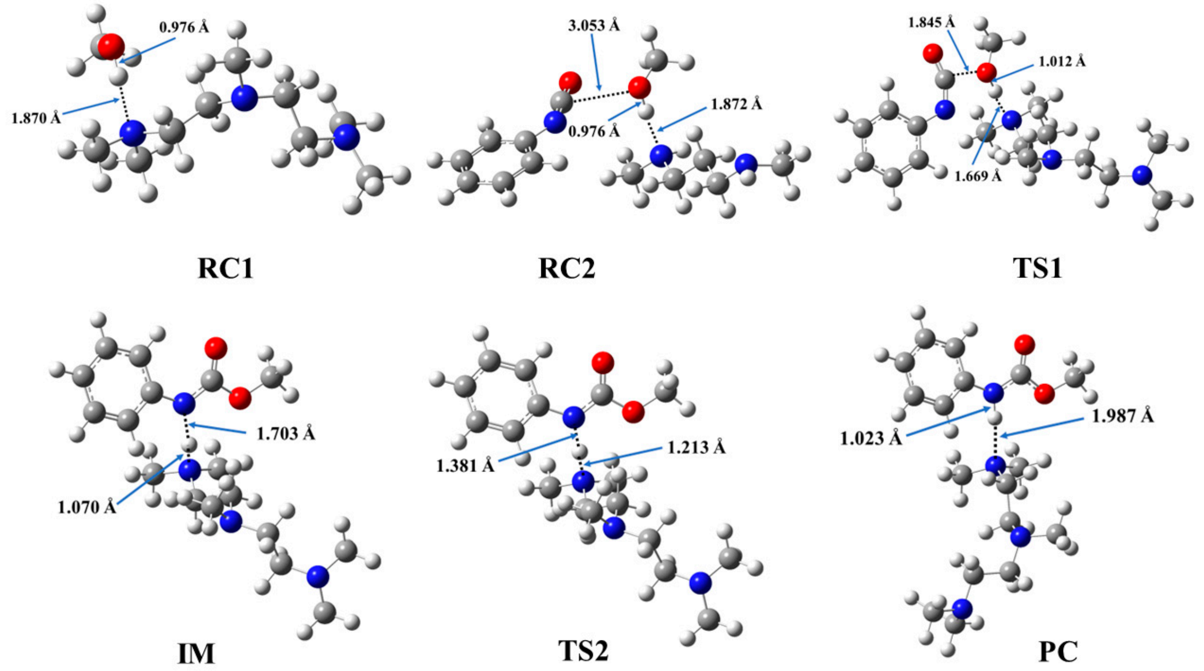

Figure S6. Optimized structures along the reaction pathway between phenyl isocyanate (PhNCO) and methanol in the presence of catalyst **7** calculated at the BHandHLYP/6-31G(d) level of theory in acetonitrile at 298.15 K and 1 atm. RC – reactant complex, TS – transition state, IM – intermediate, PC – product complex.

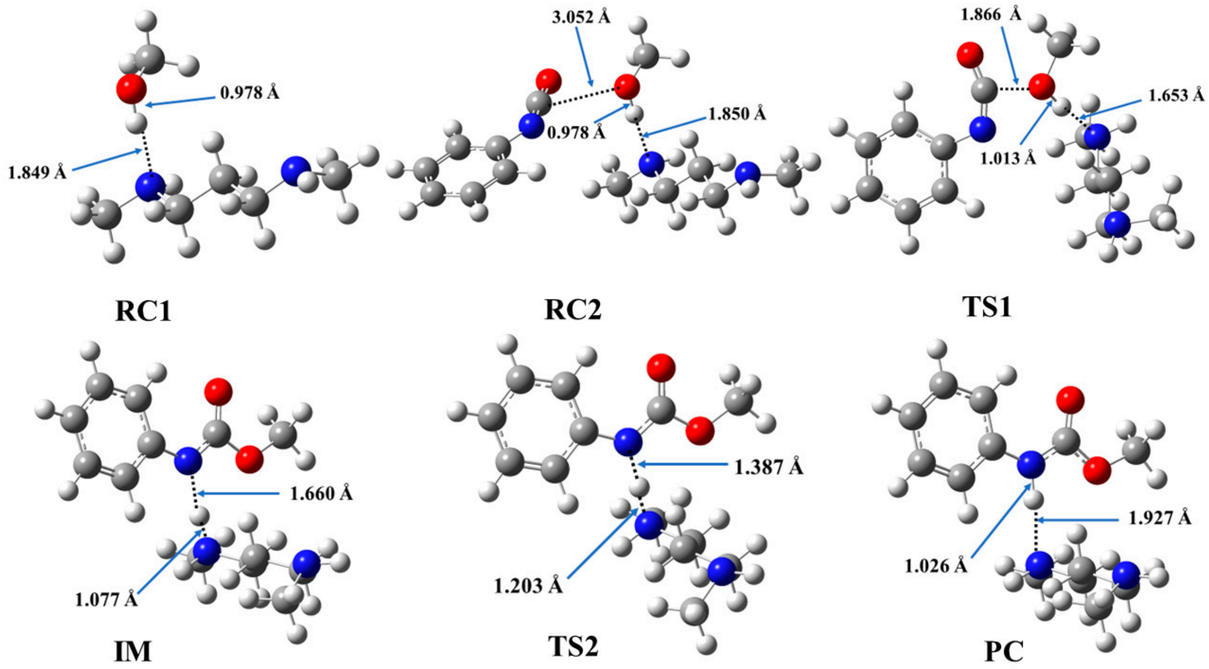

Figure S7. Optimized structures along the reaction pathway between phenyl isocyanate (PhNCO) and methanol in the presence of catalyst **8** calculated at the BHandHLYP/6-31G(d) level of theory in acetonitrile at 298.15 K and 1 atm. RC – reactant complex, TS – transition state, IM – intermediate, PC – product complex.

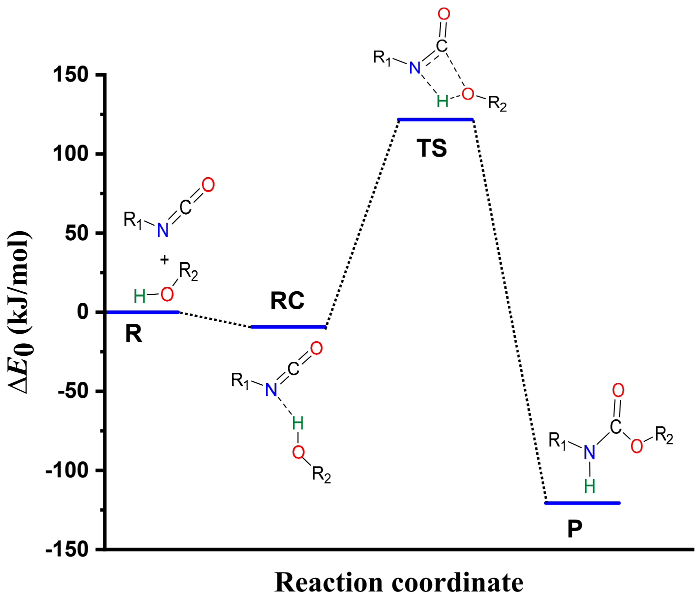

Figure S8. Energy profile (zero-point corrected, ΔE0) of the phenyl isocyanate (PhNCO) and methanol reaction calculated at the BHandHLYP/6-31G(d) level of theory in acetonitrile using the SMD implicit solvent model at 298.15 K and 1 atm.

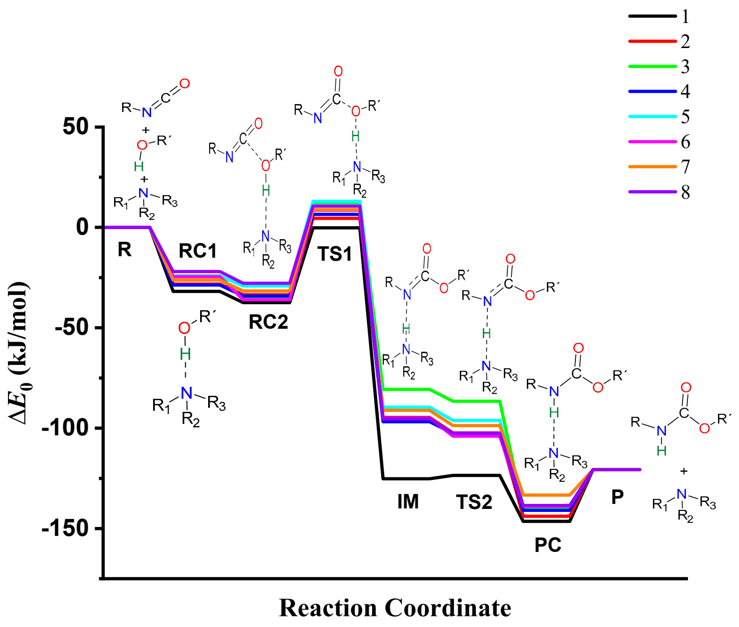

Figure S9. Energy profile (zero-point corrected, ΔE0) of the catalyzed urethane formation reactions calculated at the BHandHLYP/6-31G(d) level of theory in acetonitrile using the SMD implicit solvent model at 298.15 K and 1 atm.

Table S1. Computed and measured proton affinities (**PA**) in kJ/mol. The calculations have been carried out at the BHandHLYP level of theory in gas phase at 298.15 K and 1 atm. **RNH_2, R_2NH, R_3N,** and **$R_2C=N-R$**: primary, secondary, tertiary amines, and secondary ketimine, respectively. •side, •• middle amine group.

Catalysts	PA_{calc}				PA_{exp}[29]
	RNH_2	R_2NH	R_3N	$R_2C=N-R$	
**1**	-	-	916.7	1065.4	-

**2**	898.5	987.5	776.3	-	-
**3**	-	975.8	760.6	-	959.6
**4**	-	-	983.5	-	983.6
**5**	-	-	969.9	-	-
**6**	-	-	972.4	-	963.4
**7**	-	-	969.3•<br>965.1••	-	-
**8**	-	954.0	-	-	1035.2

Table S2. Zero-point corrected relative energies (ΔE_0), relative enthalpies (Δ***H***), and Gibbs free energies (Δ*G*) of the species in the reaction between phenyl isocyanate (PhNCO) and methanol with and without catalysts, calculated at the BHandHLYP/6-31G(d) level of theory in acetonitrile using the SMD implicit solvent model at 298.15 K and 1 atm. Cat. – catalyst, R – reactant, RC – reactant complex, TS – transition state, IM – intermediate, PC – product complex, P – product.

	**ΔE_0 (kJ/mol)**							
	**R**	**RC1**	**RC2**	**TS1**	**IM**	**TS2**	**PC**	**P**
**Cat.-free**	0.00	-	-9.36*	121.87	-	-	-	-120.67
**Cat.1**	0.00	-31.81	-37.43	-0.19	-125.23	-123.51	-146.44	-120.67
**Cat.2**	0.00	-28.22	-33.55	4.45	-94.59	-102.39	-143.83	-120.67
**Cat.3**	0.00	-24.06	-35.61	12.35	-80.63	-86.60	-140.27	-120.67
**Cat.4**	0.00	-28.72	-34.24	6.51	-96.81	-102.68	-140.90	-120.67
**Cat.5**	0.00	-24.13	-29.11	12.99	-89.53	-96.15	-138.47	-120.67
**Cat.6**	0.00	-24.51	-36.02	8.56	-94.99	-104.00	-138.83	-120.67
**Cat.7**	0.00	-26.16	-31.66	8.88	-91.15	-98.69	-133.29	-120.67
**Cat.8**	0.00	-22.03	-27.77	10.72	-95.35	-102.65	-138.53	-120.67
	**Δ*H* (kJ/mol)**							
	**R**	**RC1**	**RC2**	**TS1**	**IM**	**TS2**	**PC**	**P**
**Cat.-free**	0.00	-	-5.32*	120.36	-	-	-	-121.89
**Cat.1**	0.00	-31.03	-31.09	1.41	-124.70	-123.70	-145.15	-121.89
**Cat.2**	0.00	-27.28	-27.39	5.87	-95.06	-103.95	-143.10	-121.89
**Cat.3**	0.00	-22.90	-29.10	13.55	-80.95	-88.19	-138.95	-121.89

**Cat.4**	0.00	-28.24	-28.14	7.90	-96.69	-103.52	-140.04	-121.89
**Cat.5**	0.00	-23.37	-22.60	14.21	-89.50	-97.02	-137.66	-121.89
**Cat.6**	0.00	-23.60	-29.75	9.99	-94.64	-104.39	-137.64	-121.89
**Cat.7**	0.00	-25.32	-25.32	10.01	-91.16	-99.41	-132.46	-121.89
**Cat.8**	0.00	-21.49	-21.46	11.83	-95.54	-103.89	-137.78	-121.89
				**Δ*G* (kJ/mol)**				
	**R**	**RC1**	**RC2**	**TS1**	**IM**	**TS2**	**PC**	**P**
**Cat.-free**	0.00	-	27.84*	171.78	-	-	-	-71.72
**Cat.1**	0.00	7.60	44.58	93.02	-28.85	-26.12	-54.47	-71.72
**Cat.2**	0.00	5.95	45.17	92.13	1.39	-6.63	-53.18	-71.72
**Cat.3**	0.00	10.95	40.93	104.40	17.23	11.13	-50.86	-71.72
**Cat.4**	0.00	12.92	47.73	100.66	2.46	-0.61	-44.01	-71.72
**Cat.5**	0.00	14.71	49.51	106.38	9.17	5.09	-42.77	-71.72
**Cat.6**	0.00	11.75	43.16	99.51	1.35	-6.74	-46.93	-71.72
**Cat.7**	0.00	11.06	47.71	103.40	7.30	1.10	-38.48	-71.72
**Cat.8**	0.00	15.49	46.44	101.26	-0.19	-4.74	-46.91	-71.72

* RC for catalyst-free reaction

Table S3. Relative enthalpies (Δ***H***), and Gibbs free energies (Δ***G***) of the species in the reaction between phenyl isocyanate (PhNCO) and methanol with and without catalysts, calculated at the G3MP2BHandHLYP level of theory in acetonitrile using the SMD implicit solvent model at 298.15 K and 1 atm. Cat. – catalyst, R – reactant, RC – reactant complex, TS – transition state, IM – intermediate, PC – product complex, P – product.

				**Δ*H* (kJ/mol)**				
	**R**	**RC1**	**RC2**	**TS1**	**IM**	**TS2**	**PC**	**P**
**Cat.-free**	0.00	-	-6.65*	116.19	-	-	-	-94.03
**Cat.1**	0.00	-26.65	-36.84	-3.07	-114.92	-119.39	-126.33	-94.03
**Cat.2**	0.00	-15.32	-22.27	16.67	-77.74	-87.00	-113.12	-94.03
**Cat.3**	0.00	-18.83	-25.01	15.06	-68.43	-76.75	-113.90	-94.03
**Cat.4**	0.00	-26.35	-35.16	-3.07	-102.27	-112.89	-127.46	-94.03
**Cat.5**	0.00	-25.01	-34.70	2.58	-95.00	-106.62	-124.98	-94.03
**Cat.6**	0.00	-24.94	-33.37	1.81	-94.66	-106.20	-121.82	-94.03
**Cat.7**	0.00	-26.21	-35.90	-2.58	-98.79	-109.85	-118.24	-94.03
**Cat.8**	0.00	-18.52	-26.63	8.24	-91.59	-101.26	-119.00	-94.03
				**Δ*G* (kJ/mol)**				
	**R**	**RC1**	**RC2**	**TS1**	**IM**	**TS2**	**PC**	**P**
**Cat.-free**	0.00	-	28.99*	170.09	-	-	-	-41.38
**Cat.1**	0.00	11.97	41.31	91.02	-16.60	-19.33	-33.17	-41.38
**Cat.2**	0.00	17.91	52.76	105.40	21.19	12.79	-20.74	-41.38
**Cat.3**	0.00	15.02	47.50	108.38	32.23	25.05	-23.33	-41.38
**Cat.4**	0.00	14.97	43.36	92.34	-0.50	-7.34	-28.79	-41.38
**Cat.5**	0.00	13.07	39.88	97.22	6.15	-2.04	-27.61	-41.38
**Cat.6**	0.00	10.41	42.02	93.80	3.80	-6.07	-28.64	-41.38

**Cat.7**	0.00	11.83	41.26	94.94	3.80	-5.20	-20.12	-41.38
**Cat.8**	0.00	18.64	43.92	100.33	6.42	0.55	-25.48	-41.38

* RC for catalyst-free reaction

Table S4. Cartesian coordinates of the stationary points for the reaction system studied. The calculated at the BHandHLYP/6-31G(d) level of theory in acetonitrile at 298.15 K and 1 atm. Cat.- catalysts, RC – reactant complex, TS – transition state, IM – intermediate, PC – product complex.

**Cat.1**			
**C**	**2.00007**	**1.30303**	**0.31316**
**C**	**2.78871**	**-0.00018**	**0.34430**
**C**	**1.95443**	**-1.25696**	**0.56527**
**C**	**0.89078**	**1.32589**	**-0.73389**
**C**	**0.89520**	**-1.52065**	**-0.51250**
**C**	**-0.39223**	**-0.75538**	**-0.33120**
**H**	**1.56117**	**1.50655**	**1.29017**
**H**	**3.32370**	**-0.10383**	**-0.60135**
**H**	**1.47291**	**-1.22151**	**1.54280**
**H**	**1.31928**	**-1.31457**	**-1.49534**
**H**	**1.26868**	**0.95105**	**-1.68375**
**H**	**2.68734**	**2.12115**	**0.09958**
**H**	**3.54884**	**0.06192**	**1.12223**
**H**	**2.62786**	**-2.11207**	**0.58687**
**H**	**0.58664**	**2.35198**	**-0.91204**
**H**	**0.61493**	**-2.56787**	**-0.50126**
**N**	**-0.31452**	**0.60582**	**-0.34930**
**N**	**-1.45757**	**-1.44424**	**-0.13941**
**C**	**-2.70440**	**-0.72608**	**0.04530**
**C**	**-2.50670**	**0.61613**	**0.72749**
**H**	**-3.43974**	**1.17044**	**0.79234**
**H**	**-2.14635**	**0.45476**	**1.74250**
**H**	**-3.37786**	**-1.35193**	**0.62824**
**H**	**-3.18968**	**-0.57396**	**-0.92233**
**C**	**-1.48612**	**1.41793**	**-0.05264**
**H**	**-1.92664**	**1.78974**	**-0.97952**
**H**	**-1.16490**	**2.28469**	**0.52006**

**Cat.1-RC1**			
**O**	**-3.45519**	**-0.91973**	**-0.61158**
**C**	**-3.80189**	**-1.20096**	**0.71369**
**H**	**-2.97169**	**-1.62368**	**1.28504**
**H**	**-4.15811**	**-0.31745**	**1.25092**
**H**	**-2.68483**	**-0.31422**	**-0.58493**
**H**	**-4.60757**	**-1.93177**	**0.71030**
**C**	**2.89188**	**-0.78369**	**0.48699**
**C**	**2.22901**	**-2.14795**	**0.34502**
**C**	**0.70486**	**-2.13422**	**0.37774**
**C**	**2.44615**	**0.22585**	**-0.56516**
**C**	**0.04526**	**-1.31177**	**-0.73664**
**C**	**-0.02289**	**0.16992**	**-0.46339**
**H**	**2.70049**	**-0.36606**	**1.47559**
**H**	**2.54964**	**-2.59163**	**-0.59907**
**H**	**0.35264**	**-1.77741**	**1.34583**
**H**	**0.56483**	**-1.49124**	**-1.67758**
**H**	**2.45043**	**-0.23584**	**-1.55072**
**H**	**3.97065**	**-0.90981**	**0.40155**
**H**	**2.59353**	**-2.80542**	**1.13335**
**H**	**0.35341**	**-3.16013**	**0.28380**
**H**	**3.15818**	**1.04294**	**-0.60822**
**H**	**-0.97922**	**-1.63772**	**-0.87289**
**N**	**1.14833**	**0.83449**	**-0.30186**
**N**	**-1.19435**	**0.69628**	**-0.37100**
**C**	**-1.29221**	**2.11905**	**-0.10351**
**C**	**-0.14896**	**2.61352**	**0.76346**
**H**	**-0.19271**	**3.69106**	**0.89867**
**H**	**-0.21757**	**2.15276**	**1.74760**
**H**	**-2.24873**	**2.31096**	**0.37733**
**H**	**-1.30216**	**2.67159**	**-1.04590**
**C**	**1.16337**	**2.23262**	**0.11140**
**H**	**1.35760**	**2.86982**	**-0.75225**
**H**	**1.98742**	**2.37135**	**0.80660**

**Cat.1-RC2**			
**O**	**0.12712**	**2.68823**	**-0.63802**
**C**	**2.95189**	**2.11272**	**0.30768**
**C**	**-0.42039**	**3.65307**	**0.21421**
**H**	**-1.51275**	**3.62034**	**0.23568**
**H**	**-0.06372**	**3.55643**	**1.24222**
**C**	**4.84505**	**-2.57177**	**-0.75838**
**C**	**5.52230**	**-1.48600**	**-0.22024**
**C**	**3.49629**	**-2.45804**	**-1.06180**

**Cat.1-TS1**			
**O**	**0.47720**	**2.08470**	**-0.01712**
**C**	**2.30947**	**1.70224**	**0.20121**
**C**	**-0.05137**	**3.10725**	**0.80029**
**H**	**-0.26480**	**2.74644**	**1.80447**
**H**	**0.68162**	**3.90288**	**0.85939**
**C**	**5.87439**	**-1.70694**	**-0.60390**
**C**	**5.93464**	**-0.44166**	**-0.03840**
**C**	**4.65373**	**-2.18940**	**-1.05523**

C	2.82454	-1.26837	-0.83146	C	3.51047	-1.41702	-0.94150
C	3.50876	-0.18540	-0.29355	C	3.56503	-0.14508	-0.37295
C	4.86176	-0.29204	0.01464	C	4.79444	0.33785	0.07857
H	5.38256	0.55382	0.43331	H	4.85938	1.31826	0.51960
H	6.57047	-1.56624	0.01885	H	6.87637	-0.05334	0.31632
H	2.96269	-3.29608	-1.48006	H	4.58958	-3.17101	-1.49746
H	1.77727	-1.16507	-1.06249	H	2.55932	-1.78571	-1.28993
N	2.80049	0.99328	-0.07667	N	2.35262	0.56239	-0.29841
O	3.00713	3.21759	0.67553	O	2.76195	2.66203	0.71413
H	-0.14873	1.81075	-0.30116	H	-0.08233	1.23666	0.09125
H	-0.12524	4.63535	-0.14719	H	-0.96420	3.49929	0.35841
H	5.36518	-3.49829	-0.93914	H	6.76510	-2.30777	-0.69145
C	-5.04136	-1.04423	-0.25019	C	-5.33265	-0.59624	-0.39795
C	-5.03390	0.06484	-1.29505	C	-5.18111	0.71723	-1.15585
C	-3.87024	1.04351	-1.19051	C	-3.89407	1.48329	-0.87328
C	-3.76647	-1.88093	-0.23667	C	-4.18791	-1.57613	-0.63158
C	-2.48127	0.41208	-1.35139	C	-2.60857	0.72354	-1.22465
C	-1.93781	-0.22460	-0.09701	C	-2.15817	-0.25192	-0.16846
H	-5.19812	-0.62980	0.74587	H	-5.42201	-0.41195	0.67273
H	-5.01195	-0.39262	-2.28564	H	-5.22918	0.50709	-2.22556
H	-3.91490	1.57961	-0.24229	H	-3.85816	1.78766	0.17275
H	-2.50451	-0.31871	-2.15947	H	-2.73806	0.20419	-2.17342
H	-3.48667	-2.15024	-1.25318	H	-3.95343	-1.63332	-1.69242
H	-5.88156	-1.70786	-0.45150	H	-6.25831	-1.07579	-0.71371
H	-5.96933	0.61951	-1.23290	H	-6.03137	1.35911	-0.92934
H	-3.98508	1.79329	-1.97122	H	-3.90686	2.40013	-1.45947
H	-3.95038	-2.81289	0.28693	H	-4.49591	-2.57185	-0.33242
H	-1.76653	1.17643	-1.63266	H	-1.79428	1.42555	-1.36166
N	-2.64112	-1.25093	0.44200	N	-2.98051	-1.28070	0.13209
N	-0.85914	0.27731	0.39681	N	-1.02578	-0.02244	0.40554
C	-0.32366	-0.30483	1.61398	C	-0.55771	-0.94081	1.42742
C	-1.42148	-0.83202	2.51962	C	-1.71153	-1.53788	2.20998
H	-1.00862	-1.32434	3.39627	H	-1.36156	-2.27913	2.92333
H	-2.03533	-0.00106	2.86360	H	-2.21442	-0.75020	2.76842
H	0.26498	0.45428	2.12395	H	0.11798	-0.40031	2.08551
H	0.36500	-1.11500	1.36475	H	0.02819	-1.73737	0.96492
C	-2.27519	-1.80853	1.73853	C	-2.68338	-2.17967	1.24272
H	-1.74294	-2.75002	1.59752	H	-2.27589	-3.11432	0.85684
H	-3.19037	-2.03268	2.28056	H	-3.61840	-2.41556	1.74352
**Cat.1-IM**				**Cat.1-TS2**			
O	0.46849	2.11832	0.89186	O	0.39123	2.21547	0.82408
C	1.74162	1.74482	0.55184	C	1.64057	1.82008	0.47410
C	0.30208	3.46494	1.27268	C	0.24524	3.58288	1.15063
H	-0.74459	3.57806	1.53277	H	-0.79534	3.71466	1.42254
H	0.91670	3.71687	2.13213	H	0.87834	3.85879	1.98816
C	5.13768	-1.75177	-0.97220	C	4.95180	-1.82252	-0.94102
C	5.29870	-0.43823	-0.55862	C	5.15514	-0.53256	-0.47641
C	3.85387	-2.28421	-0.98706	C	3.64969	-2.29704	-1.02867
C	2.77046	-1.51997	-0.59811	C	2.58236	-1.49851	-0.66116
C	2.91062	-0.18463	-0.17296	C	2.77378	-0.19114	-0.18658
C	4.21779	0.33830	-0.16631	C	4.09296	0.27745	-0.10194

H	4.36906	1.35308	0.14709	H	4.27380	1.27376	0.25187
H	6.28625	-0.00125	-0.53822	H	6.15859	-0.14204	-0.39916
H	3.69461	-3.30350	-1.30540	H	3.46066	-3.29644	-1.38939
H	1.77971	-1.94695	-0.61971	H	1.57785	-1.88070	-0.74350
N	1.74904	0.46268	0.20286	N	1.63389	0.51423	0.18165
O	2.63879	2.58003	0.60372	O	2.55957	2.62260	0.45291
H	0.17893	-0.35404	0.39197	H	0.49797	-0.13538	0.32268
H	0.54729	4.14425	0.46074	H	0.48404	4.21968	0.30426
H	5.98587	-2.34477	-1.27498	H	5.78518	-2.44286	-1.22923
C	-4.66105	0.38584	-0.85946	C	-4.59951	0.21380	-0.84692
C	-3.87560	1.46615	-1.59281	C	-3.86425	1.21360	-1.73074
C	-2.43326	1.64214	-1.13336	C	-2.41134	1.46975	-1.34832
C	-4.03322	-0.99871	-0.95127	C	-3.93373	-1.15555	-0.78346
C	-1.55235	0.39740	-1.30011	C	-1.50771	0.23147	-1.40130
C	-1.73780	-0.61933	-0.21327	C	-1.60650	-0.64837	-0.18548
H	-4.78597	0.64671	0.19111	H	-4.70276	0.60121	0.16653
H	-3.87437	1.23153	-2.65807	H	-3.89230	0.85241	-2.75991
H	-2.40053	1.96803	-0.09519	H	-2.35064	1.91442	-0.35614
H	-1.74967	-0.07550	-2.26048	H	-1.73484	-0.35523	-2.29012
H	-3.70847	-1.20518	-1.96762	H	-3.64336	-1.48231	-1.77913
H	-5.65887	0.32558	-1.29052	H	-5.60731	0.07832	-1.23671
H	-4.39566	2.41666	-1.48592	H	-4.40304	2.15991	-1.72090
H	-1.97829	2.43720	-1.71960	H	-1.99940	2.20279	-2.03880
H	-4.76415	-1.75567	-0.69569	H	-4.63858	-1.88933	-0.41033
H	-0.50894	0.68626	-1.28947	H	-0.47326	0.54140	-1.48146
N	-2.91040	-1.20282	-0.03109	N	-2.77984	-1.21686	0.11238
N	-0.70805	-0.87564	0.55248	N	-0.53871	-0.78574	0.53933
C	-0.74996	-1.80762	1.66551	C	-0.55344	-1.58834	1.74780
C	-2.15900	-1.85976	2.21734	C	-1.92725	-1.56118	2.38680
H	-2.24697	-2.64690	2.95932	H	-1.97496	-2.23882	3.23416
H	-2.39663	-0.91436	2.69956	H	-2.13920	-0.55702	2.74841
H	-0.04119	-1.46056	2.40918	H	0.20188	-1.18807	2.41832
H	-0.43044	-2.79254	1.33110	H	-0.26757	-2.61385	1.51425
C	-3.13697	-2.11982	1.09021	C	-2.96192	-1.96312	1.35676
H	-3.05191	-3.14287	0.72946	H	-2.90221	-3.03035	1.14752
H	-4.15573	-1.96959	1.42985	H	-3.96262	-1.75524	1.72213
**Cat.1-PC**							
O	0.64512	2.38825	0.43457				
C	1.86723	1.90978	0.17266				
C	0.53823	3.80275	0.51341				
H	-0.50367	4.00477	0.72576				
H	1.16114	4.19388	1.31049				
C	4.92248	-2.19116	-0.55543				
C	5.20321	-0.83454	-0.56425				
C	3.61791	-2.60354	-0.32105				
C	2.61621	-1.67630	-0.09957				
C	2.89972	-0.30798	-0.10784				
C	4.20964	0.10882	-0.34419				
H	4.44284	1.15556	-0.35418				
H	6.21098	-0.49449	-0.74466				
H	3.37478	-3.65428	-0.30951				

**H**	**1.60484**	**-2.00138**	**0.08259**
**N**	**1.82765**	**0.55910**	**0.12722**
**O**	**2.83330**	**2.62106**	**0.00863**
**H**	**0.91203**	**0.12427**	**0.29482**
**H**	**0.82121**	**4.26517**	**-0.42588**
**H**	**5.70334**	**-2.91401**	**-0.72747**
**C**	**-4.60613**	**0.09668**	**-1.16612**
**C**	**-3.73363**	**0.40525**	**-2.37646**
**C**	**-2.24817**	**0.57611**	**-2.07900**
**C**	**-4.17176**	**-1.14837**	**-0.39960**
**C**	**-1.57086**	**-0.65512**	**-1.46357**
**C**	**-1.76317**	**-0.79106**	**0.02575**
**H**	**-4.61983**	**0.94286**	**-0.47894**
**H**	**-3.84877**	**-0.40307**	**-3.10058**
**H**	**-2.08916**	**1.43596**	**-1.42817**
**H**	**-1.91997**	**-1.55486**	**-1.96941**
**H**	**-3.96456**	**-1.96090**	**-1.09296**
**H**	**-5.63115**	**-0.05169**	**-1.50404**
**H**	**-4.10311**	**1.30787**	**-2.86148**
**H**	**-1.73817**	**0.80190**	**-3.01373**
**H**	**-4.98519**	**-1.48729**	**0.23295**
**H**	**-0.50033**	**-0.59852**	**-1.62436**
**N**	**-3.03127**	**-0.94105**	**0.48329**
**N**	**-0.71114**	**-0.71872**	**0.76423**
**C**	**-0.87414**	**-0.82339**	**2.20230**
**C**	**-2.19077**	**-0.22914**	**2.66768**
**H**	**-2.33235**	**-0.36698**	**3.73645**
**H**	**-2.19198**	**0.84108**	**2.46721**
**H**	**-0.03565**	**-0.31741**	**2.67622**
**H**	**-0.81743**	**-1.87103**	**2.50658**
**C**	**-3.32218**	**-0.89283**	**1.91143**
**H**	**-3.48898**	**-1.90305**	**2.28706**
**H**	**-4.24782**	**-0.33997**	**2.04953**

**Cat.2**				**Cat.2-RC1**			
**C**	**-1.83990**	**-1.05366**	**-0.20526**	**C**	**-5.49711**	**0.82299**	**-0.18007**
**C**	**-2.86213**	**0.80926**	**-0.22848**	**H**	**-4.84553**	**1.68426**	**-0.00746**
**C**	**-1.63852**	**1.05344**	**0.31448**	**H**	**-5.62645**	**0.71700**	**-1.26099**
**N**	**-0.99002**	**-0.15208**	**0.32629**	**H**	**-6.47022**	**1.05553**	**0.24669**
**H**	**-1.56968**	**-2.08797**	**-0.30835**	**O**	**-5.01969**	**-0.34377**	**0.42306**
**H**	**-3.66128**	**1.50649**	**-0.39945**	**H**	**-4.14085**	**-0.53058**	**0.03797**
**H**	**-1.17934**	**1.94677**	**0.68977**	**C**	**3.11095**	**1.17767**	**-0.11383**
**N**	**-2.98090**	**-0.51628**	**-0.55202**	**C**	**4.31044**	**-0.57185**	**-0.24241**
**C**	**0.36546**	**-0.40319**	**0.78501**	**C**	**3.10847**	**-0.97052**	**0.25571**
**H**	**0.46585**	**-1.47610**	**0.91248**	**N**	**2.34527**	**0.16313**	**0.33578**
**H**	**0.48228**	**0.05633**	**1.76233**	**H**	**2.74247**	**2.18574**	**-0.15133**
**C**	**1.41793**	**0.12134**	**-0.17966**	**H**	**5.17645**	**-1.17479**	**-0.44357**
**H**	**1.29683**	**1.19505**	**-0.30972**	**H**	**2.73342**	**-1.92804**	**0.55934**
**H**	**1.26811**	**-0.34057**	**-1.15552**	**N**	**4.30394**	**0.77838**	**-0.47179**
**C**	**2.82715**	**-0.16699**	**0.30963**	**C**	**0.96529**	**0.24972**	**0.78148**
**H**	**2.93726**	**-1.24303**	**0.47630**	**H**	**0.75678**	**1.29715**	**0.97462**

**H**	**2.98147**	**0.31604**	**1.27310**	**H**	**0.87995**	**-0.28227**	**1.72458**
**N**	**3.80885**	**0.37007**	**-0.62515**	**C**	**-0.01737**	**-0.30999**	**-0.23677**
**H**	**4.73766**	**0.20017**	**-0.26693**	**H**	**0.21708**	**-1.35576**	**-0.43261**
**H**	**3.75544**	**-0.14641**	**-1.49222**	**H**	**0.09367**	**0.22811**	**-1.17757**
				**C**	**-1.45666**	**-0.19826**	**0.24798**
				**H**	**-1.70138**	**0.84477**	**0.43857**
				**H**	**-1.56973**	**-0.72571**	**1.19299**
				**N**	**-2.44971**	**-0.72850**	**-0.68100**
				**H**	**-2.25452**	**-1.70227**	**-0.86969**
				**H**	**-2.37414**	**-0.25261**	**-1.56957**
**Cat.2-RC2**				**Cat.2-TS1**			
**C**	**2.33455**	**-3.11695**	**-0.58404**	**C**	**2.93308**	**-3.29476**	**-0.12472**
**C**	**3.35551**	**-3.81332**	**0.04539**	**C**	**4.29917**	**-3.47325**	**0.04287**
**C**	**4.42512**	**-3.11778**	**0.59264**	**C**	**5.11995**	**-2.35916**	**0.13903**
**C**	**4.47861**	**-1.73662**	**0.51453**	**C**	**4.59063**	**-1.07994**	**0.07009**
**C**	**3.45023**	**-1.04418**	**-0.11808**	**C**	**3.21685**	**-0.89668**	**-0.09795**
**C**	**2.37774**	**-1.73449**	**-0.66851**	**C**	**2.39697**	**-2.02028**	**-0.19460**
**H**	**1.50052**	**-3.64846**	**-1.01286**	**H**	**2.28057**	**-4.15010**	**-0.20155**
**H**	**3.31946**	**-4.88857**	**0.10932**	**H**	**4.71759**	**-4.46520**	**0.09741**
**H**	**5.22351**	**-3.65076**	**1.08325**	**H**	**6.18366**	**-2.48215**	**0.26914**
**H**	**5.30725**	**-1.19247**	**0.93808**	**H**	**5.24294**	**-0.22649**	**0.14614**
**H**	**1.59217**	**-1.18374**	**-1.15887**	**H**	**1.33664**	**-1.87668**	**-0.32505**
**N**	**3.45888**	**0.34632**	**-0.22026**	**N**	**2.58215**	**0.35555**	**-0.18040**
**C**	**4.14396**	**1.27064**	**0.10528**	**C**	**3.24446**	**1.40793**	**-0.08837**
**O**	**4.75338**	**2.22453**	**0.37950**	**O**	**4.23257**	**2.03622**	**0.04927**
**C**	**1.94386**	**4.11719**	**0.36113**	**C**	**2.14639**	**3.97017**	**0.18583**
**H**	**1.00951**	**4.67971**	**0.44529**	**H**	**1.43372**	**4.67248**	**-0.23890**
**H**	**2.16356**	**3.68940**	**1.34219**	**H**	**2.08346**	**4.00262**	**1.27161**
**H**	**2.73520**	**4.82415**	**0.12445**	**H**	**3.14674**	**4.24962**	**-0.12051**
**O**	**1.89787**	**3.15292**	**-0.65042**	**O**	**1.88503**	**2.67244**	**-0.31093**
**H**	**1.22684**	**2.49091**	**-0.39130**	**H**	**1.02570**	**2.31884**	**0.08872**
**C**	**-5.87947**	**0.28986**	**0.58392**	**C**	**-5.91945**	**0.16016**	**-0.46678**
**C**	**-6.09417**	**-1.79523**	**0.23406**	**C**	**-6.22999**	**-1.62695**	**0.64070**
**C**	**-4.97045**	**-1.41172**	**-0.43069**	**C**	**-4.89653**	**-1.57085**	**0.37548**
**N**	**-4.84276**	**-0.06839**	**-0.20014**	**N**	**-4.70810**	**-0.41814**	**-0.33852**
**H**	**-6.01261**	**1.30447**	**0.90995**	**H**	**-6.04699**	**1.08645**	**-0.99511**
**H**	**-6.52881**	**-2.77612**	**0.28808**	**H**	**-6.76675**	**-2.38785**	**1.17633**
**H**	**-4.26887**	**-1.95126**	**-1.03601**	**H**	**-4.08328**	**-2.22709**	**0.61560**
**N**	**-6.65923**	**-0.72100**	**0.86847**	**N**	**-6.86520**	**-0.53661**	**0.10823**
**C**	**-3.77542**	**0.79520**	**-0.67495**	**C**	**-3.44288**	**0.10073**	**-0.82799**
**H**	**-4.11210**	**1.81910**	**-0.54863**	**H**	**-3.67238**	**0.85484**	**-1.57366**
**H**	**-3.64655**	**0.62021**	**-1.73922**	**H**	**-2.91644**	**-0.70697**	**-1.32812**
**C**	**-2.46456**	**0.57110**	**0.06452**	**C**	**-2.58373**	**0.69233**	**0.27989**
**H**	**-2.14793**	**-0.46387**	**-0.06009**	**H**	**-2.37169**	**-0.07311**	**1.02537**
**H**	**-2.61993**	**0.73585**	**1.13025**	**H**	**-3.13685**	**1.48689**	**0.77927**
**C**	**-1.36757**	**1.49942**	**-0.43967**	**C**	**-1.27441**	**1.24635**	**-0.26240**
**H**	**-1.67548**	**2.53542**	**-0.31278**	**H**	**-1.47443**	**2.02022**	**-0.99999**
**H**	**-1.21457**	**1.34497**	**-1.50591**	**H**	**-0.71622**	**0.46167**	**-0.76781**
**N**	**-0.08008**	**1.33004**	**0.22612**	**N**	**-0.40107**	**1.82040**	**0.75997**
**H**	**0.24510**	**0.38025**	**0.10772**	**H**	**-0.18471**	**1.12195**	**1.45839**
**H**	**-0.19148**	**1.46373**	**1.22199**	**H**	**-0.87967**	**2.56779**	**1.24462**

Cat.2-IM				Cat.2-TS2			
O	-1.93393	-2.57234	-0.24999	O	-2.19220	-2.50257	-0.24160
C	-2.46525	-1.34888	-0.56171	C	-2.58997	-1.23392	-0.54806
C	-2.37321	-3.65764	-1.03868	C	-2.74963	-3.53411	-1.03041
H	-1.85890	-4.53252	-0.65730	H	-2.33033	-4.45883	-0.65134
H	-3.44613	-3.80172	-0.95364	H	-3.83115	-3.56043	-0.93982
C	-2.87643	3.67805	0.26788	C	-2.41791	3.81268	0.28072
C	-3.43948	2.86049	-0.69991	C	-3.00919	3.07027	-0.72970
C	-2.02812	3.10248	1.20583	C	-1.70561	3.14008	1.26523
C	-1.75561	1.74801	1.17048	C	-1.59158	1.76263	1.23486
C	-2.31707	0.90220	0.19685	C	-2.18358	0.99542	0.21751
C	-3.17307	1.49966	-0.74492	C	-2.90103	1.68791	-0.77102
H	-3.62180	0.88695	-1.50266	H	-3.36889	1.13362	-1.56134
H	-4.10187	3.28459	-1.43983	H	-3.56759	3.57102	-1.50627
H	-1.57456	3.71281	1.97199	H	-1.23552	3.69086	2.06551
H	-1.09526	1.31730	1.90751	H	-1.03865	1.25906	2.01225
N	-1.96317	-0.43391	0.26283	N	-1.98913	-0.37626	0.27809
O	-3.26917	-1.26104	-1.48022	O	-3.38306	-1.04748	-1.45796
H	-0.76820	-2.41809	2.33725	H	-0.94751	-2.39660	2.27069
H	-2.12121	-3.51592	-2.08542	H	-2.48711	-3.41736	-2.07738
H	-3.09133	4.73435	0.29259	H	-2.50922	4.88674	0.30261
C	4.97877	0.31898	0.23169	C	4.92402	0.10988	0.24938
C	4.54969	1.67053	-1.35160	C	4.57328	1.46392	-1.35095
C	3.53571	0.76377	-1.34211	C	3.53010	0.59074	-1.35735
N	3.82255	-0.10227	-0.32076	N	3.76683	-0.27723	-0.32505
H	5.42679	-0.19764	1.05980	H	5.33708	-0.41520	1.09027
H	4.68415	2.50561	-2.01369	H	4.74920	2.28953	-2.01520
H	2.66119	0.64983	-1.95196	H	2.66504	0.50153	-1.98453
N	5.45131	1.38430	-0.36066	N	5.44396	1.15507	-0.33936
C	3.02353	-1.23596	0.10410	C	2.91875	-1.37806	0.09198
H	3.65061	-1.85179	0.74009	H	3.51451	-2.01964	0.73274
H	2.76618	-1.82073	-0.77396	H	2.64684	-1.95082	-0.78963
C	1.76508	-0.81044	0.84726	C	1.67152	-0.90203	0.82309
H	1.15106	-0.18809	0.20054	H	1.10060	-0.23918	0.17701
H	2.04232	-0.21425	1.71502	H	1.96557	-0.33159	1.70279
C	0.97129	-2.02380	1.29072	C	0.80831	-2.07757	1.23934
H	1.54988	-2.64773	1.96341	H	1.35546	-2.74715	1.89592
H	0.65787	-2.62180	0.44315	H	0.47478	-2.64151	0.37515
N	-0.26338	-1.60864	1.99187	N	-0.40539	-1.60884	1.93499
H	-0.94253	-1.07032	1.32599	H	-1.10536	-1.00173	1.22342
H	-0.04953	-1.02478	2.79460	H	-0.16457	-1.04791	2.74512

Cat.2-PC			
O	-1.73600	-2.63595	-0.31001
C	-2.35558	-1.50248	-0.66322
C	-2.02212	-3.77402	-1.11165
H	-1.44673	-4.58433	-0.68317
H	-3.07948	-4.01285	-1.07949
C	-3.19012	3.51231	0.25804
C	-3.65322	2.68398	-0.75143
C	-2.34185	2.98435	1.22152
C	-1.96652	1.65433	1.17423

C	-2.43271	0.81933	0.15589
C	-3.28481	1.34772	-0.81356
H	-3.65180	0.71924	-1.60147
H	-4.31311	3.07617	-1.50934
H	-1.96843	3.60909	2.01740
H	-1.30810	1.25153	1.92675
N	-1.99775	-0.50919	0.18110
O	-3.11275	-1.42888	-1.60461
H	-0.59796	-2.16603	2.67864
H	-1.72004	-3.60802	-2.13992
H	-3.48346	4.54881	0.29490
C	4.93797	0.67172	0.19891
C	4.43628	1.59756	-1.64700
C	3.52657	0.60287	-1.46067
N	3.85996	0.01259	-0.27141
H	5.40245	0.39846	1.12795
H	4.50883	2.28221	-2.47161
H	2.69676	0.26244	-2.04825
N	5.31906	1.63427	-0.60043
C	3.16363	-1.08557	0.37605
H	3.83345	-1.49141	1.12718
H	2.99239	-1.86047	-0.36551
C	1.84975	-0.65304	1.00912
H	1.20386	-0.21434	0.25092
H	2.04878	0.11966	1.75136
C	1.13287	-1.82052	1.66540
H	1.79875	-2.29938	2.38727
H	0.88457	-2.56583	0.91372
N	-0.11695	-1.37434	2.27564
H	-1.34722	-0.77008	0.93054
H	0.08546	-0.75084	3.04497

Cat.3				Cat.3-RC1			
C	0.18984	-1.07612	0.00001	N	-0.27943	0.19014	-0.31711
C	1.49321	0.60223	-0.00000	H	-2.11784	-0.07257	-0.62252
C	0.22767	1.10290	0.00001	C	0.35057	1.37837	-0.05852
N	-0.60100	0.01461	-0.00000	C	-3.52196	-0.30229	0.67044
H	-0.22130	-2.06820	0.00001	H	-3.32890	0.60914	1.24246
H	2.41981	1.14569	-0.00001	H	-3.06413	-1.13598	1.20938
H	-0.15506	2.10460	0.00001	H	-4.59706	-0.46514	0.65941
N	1.46091	-0.76680	-0.00001	C	1.67465	1.16069	0.15881
C	-2.04721	0.03476	-0.00000	N	1.85732	-0.18982	0.03035
H	-2.41009	-0.98563	-0.00001	C	0.65734	-0.72312	-0.25415
H	-2.41689	0.54316	-0.88452	H	2.48759	1.82119	0.38753
H	-2.41689	0.54314	0.88453	H	-0.18350	2.30953	-0.04566
				H	0.52455	-1.77748	-0.40527
				C	3.10761	-0.90394	0.17791
				H	2.93056	-1.95793	0.00374
				H	3.49980	-0.76921	1.18043
				H	3.83270	-0.54048	-0.54247
				O	-3.07980	-0.21019	-0.65333

Cat.3-RC2	Cat.3-TS1

C	-1.03944	1.84196	-0.46141	C	1.14407	1.71449	-0.05940
N	2.53229	-0.17292	0.26818	N	-2.29185	0.10343	-0.07542
H	0.94877	-0.04261	1.22636	H	-1.16528	1.31877	-0.40855
C	2.88494	0.68891	-0.73629	C	-2.03636	-1.07320	0.57709
C	0.47090	1.05301	2.74833	C	-1.15854	3.24176	0.11119
H	0.80419	1.99759	2.31745	H	-1.43744	3.00874	1.13568
H	1.24744	0.69466	3.42776	H	-2.03667	3.55571	-0.44543
H	-0.42315	1.24712	3.33501	H	-0.42715	4.03927	0.10859
C	-4.81516	-1.72809	-0.05620	C	4.63696	-1.85947	0.07730
C	-5.03679	-0.78714	-1.05107	C	4.68327	-0.52398	0.44925
C	-3.66142	-1.65652	0.71231	C	3.45480	-2.37908	-0.43167
C	-2.72851	-0.65514	0.49738	C	2.33677	-1.57387	-0.56563
C	-2.96205	0.28381	-0.50299	C	2.37661	-0.23037	-0.19273
C	-4.11339	0.22010	-1.27834	C	3.56797	0.28880	0.31824
H	-5.92953	-0.83440	-1.65340	H	5.59498	-0.10581	0.84615
H	-3.48346	-2.38544	1.48677	H	3.40129	-3.41520	-0.72650
H	-1.82625	-0.58917	1.08426	H	1.41655	-1.97170	-0.96184
N	-2.05875	1.30970	-0.76010	N	1.19087	0.50373	-0.36560
O	-0.07032	2.47301	-0.28831	O	1.61378	2.71706	0.35254
C	4.10360	0.34498	-1.23016	C	-3.18090	-1.79885	0.66926
N	4.50347	-0.75007	-0.51240	N	-4.15224	-1.04920	0.06080
C	3.52733	-1.01843	0.37113	C	-3.56951	0.07971	-0.36829
H	4.71263	0.76413	-2.00663	H	-3.39463	-2.75680	1.10039
H	2.24202	1.49435	-1.03502	H	-1.05453	-1.31878	0.93284
H	3.59320	-1.83779	1.06135	H	-4.11426	0.84736	-0.88330
H	-4.27406	0.95646	-2.04802	H	3.62269	1.32315	0.61221
C	5.74729	-1.47244	-0.67436	C	-5.54659	-1.41086	-0.08800
H	5.76102	-2.30615	0.01674	H	-6.06042	-0.61120	-0.60639
H	6.58919	-0.82120	-0.46409	H	-5.99991	-1.55449	0.88686
H	5.83019	-1.84932	-1.68815	H	-5.63499	-2.32567	-0.66398
O	0.15030	0.10522	1.76593	O	-0.57595	2.11992	-0.53087
H	-5.53496	-2.51107	0.11890	H	5.50842	-2.48532	0.18183
**Cat.3-IM**				**Cat.3-TS2**			
3-IM.log	Energy:	179.7630000		C	3.00574	-2.36284	-0.38851
C	-3.07895	-2.36523	0.24474	C	4.24276	-1.84735	-0.02402
C	-4.32887	-1.79812	0.03029	C	4.32514	-0.50508	0.31430
C	-4.40416	-0.42830	-0.17048	C	3.20729	0.31583	0.29528
C	-3.26939	0.36971	-0.16234	C	1.94993	-0.19173	-0.07055
C	-1.99669	-0.18903	0.05071	C	1.88518	-1.55290	-0.41217
C	-1.94179	-1.57945	0.25498	H	2.91142	-3.40305	-0.66058
H	-2.98758	-3.42843	0.40754	H	5.11870	-2.47580	-0.00641
H	-5.21810	-2.40789	0.02218	H	5.27612	-0.08229	0.60180
H	-5.36435	0.03635	-0.33864	H	3.29583	1.35276	0.55668
H	-3.35386	1.42789	-0.31714	H	0.93488	-1.96675	-0.71102
H	-0.98106	-2.03672	0.43285	N	0.75818	0.51598	-0.09762
N	-0.78876	0.48689	0.06499	C	0.71457	1.84561	-0.03703
C	-0.71777	1.81365	0.01263	O	1.60968	2.67644	0.01901
O	-1.58926	2.67160	-0.04088	C	-0.79893	3.65111	-0.00105
C	0.83660	3.58742	-0.01898	H	-1.87355	3.79285	-0.01153
H	1.91401	3.70785	-0.00624	H	-0.38525	4.07660	0.90807
H	0.43372	4.02588	-0.92697	H	-0.35813	4.15437	-0.85623

H	0.40509	4.09757	0.83705	O	-0.58815	2.25562	-0.05958
O	0.59674	2.19722	0.03392	H	-0.55973	-0.15740	-0.02305
H	0.63834	-0.22889	0.00050	N	-1.58356	-0.63892	0.05577
N	1.63201	-0.66942	-0.05153	C	-1.86022	-1.93586	0.39579
C	1.93687	-1.97706	-0.31843	C	-3.20428	-2.09114	0.36501
C	3.28379	-2.09415	-0.27686	N	-3.73134	-0.87546	0.00365
N	3.77963	-0.84670	0.01695	C	-2.72512	-0.02553	-0.17316
C	2.75617	-0.01232	0.14555	H	-3.82868	-2.93852	0.56433
H	3.92982	-2.93512	-0.42768	H	-1.08608	-2.63767	0.63134
H	1.17914	-2.70788	-0.51502	H	-2.83361	1.00183	-0.45378
H	2.83453	1.03079	0.37113	C	-5.14394	-0.57935	-0.15683
C	5.18462	-0.50566	0.16065	H	-5.25414	0.46627	-0.41296
H	5.26565	0.55321	0.36840	H	-5.66451	-0.78233	0.77177
H	5.70861	-0.73750	-0.75908	H	-5.55715	-1.19315	-0.94879
H	5.61347	-1.07176	0.97912				

Cat.3-PC

C	-2.92047	-2.50280	0.19813
C	-4.22296	-2.03002	0.11830
C	-4.43354	-0.66695	-0.01420
C	-3.37141	0.22392	-0.06955
C	-2.06394	-0.25368	0.01263
C	-1.85128	-1.62712	0.14778
H	-2.73162	-3.55943	0.30292
H	-5.05664	-2.71202	0.15907
H	-5.43885	-0.28123	-0.07868
H	-3.54988	1.27653	-0.17272
H	-0.84254	-2.00103	0.21811
N	-0.92743	0.55977	-0.04364
C	-0.88845	1.91132	-0.02921
O	-1.82965	2.67029	0.01611
C	0.57849	3.73331	-0.06940
H	1.64950	3.88004	-0.11980
H	0.10070	4.19009	-0.92897
H	0.18621	4.17484	0.84011
O	0.38408	2.32505	-0.07225
H	-0.02500	0.08561	-0.07803
N	1.75197	-0.75680	-0.15134
C	2.23736	-2.00460	-0.44012
C	3.58943	-2.01961	-0.29655
N	3.93799	-0.75325	0.08864
C	2.80183	-0.03862	0.15974
H	4.32139	-2.79183	-0.42895
H	1.58985	-2.80960	-0.73244
H	2.79217	0.99746	0.43864
C	5.27691	-0.27653	0.36191
H	5.22318	0.76538	0.65228
H	5.89596	-0.36813	-0.52431
H	5.72058	-0.84912	1.16952

Cat.4	Cat.4-RC1

C	-0.43998	-1.25039	-0.22853	O	-2.80703	-1.01802	-0.81181
H	-0.40630	-1.30418	-1.31818	N	-0.86691	0.89901	-0.01374
H	0.04152	-2.14788	0.14774	C	-1.17789	2.02889	-0.87146
C	-0.43998	1.25039	-0.22853	H	-0.55350	2.90525	-0.67592
H	0.04152	2.14788	0.14775	H	-2.21456	2.31953	-0.71929
H	-0.40630	1.30418	-1.31818	H	-1.05471	1.74602	-1.91332
C	-1.89245	1.24762	0.24116	C	-1.09593	1.24239	1.38004
H	-2.39074	2.14472	-0.12332	H	-2.14434	1.49717	1.51360
H	-1.91732	1.29960	1.33110	H	-0.50118	2.09710	1.71332
C	-1.89245	-1.24762	0.24115	H	-0.87261	0.39891	2.02426
H	-2.39074	-2.14471	-0.12332	C	0.44877	0.30612	-0.30360
H	-1.91732	-1.29960	1.33110	C	1.65450	1.13515	0.15059
N	1.69643	-0.00000	-0.31887	H	0.49188	0.23629	-1.39086
C	0.32713	-0.00000	0.21356	H	1.64615	1.22467	1.23710
H	0.36591	-0.00000	1.31432	H	1.59380	2.14473	-0.25201
C	-2.63497	0.00000	-0.21478	C	-3.61605	-1.31068	0.29253
H	-2.71054	0.00000	-1.30360	H	-4.20115	-0.44921	0.62479
C	2.44553	1.18150	0.06984	H	-4.31509	-2.09200	0.00318
H	3.46541	1.09196	-0.29353	H	-2.14092	-0.36020	-0.53169
H	2.02487	2.07899	-0.36856	H	-3.04408	-1.67772	1.14795
H	2.48880	1.32072	1.15817	C	0.54941	-1.12066	0.23423
C	2.44554	-1.18149	0.06984	H	0.50137	-1.11228	1.32320
H	2.48880	-1.32071	1.15818	H	-0.30397	-1.69448	-0.12077
H	2.02487	-2.07899	-0.36855	C	2.96396	0.47888	-0.28371
H	3.46541	-1.09196	-0.29352	H	3.01988	0.47517	-1.37370
H	-3.65315	0.00000	0.17220	H	3.80884	1.06840	0.06943
				C	1.85772	-1.78001	-0.19656
				H	1.86342	-1.89164	-1.28218
				H	1.92034	-2.78416	0.22017
				C	3.06750	-0.95384	0.22883
				H	3.12766	-0.94088	1.31856
				H	3.98545	-1.41714	-0.13047
**Cat.4-RC2**				**Cat.4-TS1**			
O	-0.46420	2.10474	-1.33917	O	-0.07058	2.27551	-0.73025
C	2.22824	1.95693	0.05665	C	1.66979	1.71606	-0.47924
N	-1.89566	0.91023	0.81122	N	-1.88861	1.03859	0.80544
C	-0.88403	0.78485	1.84603	C	-1.29036	1.04463	2.13500
H	-1.27777	0.39850	2.79028	H	-1.96801	0.67335	2.90560
H	-0.45015	1.76231	2.04331	H	-1.01192	2.06227	2.39617
H	-0.09175	0.12317	1.50853	H	-0.39338	0.43346	2.13569
C	-2.94211	1.82516	1.23679	C	-3.06809	1.89651	0.78180
H	-2.50144	2.80014	1.42928	H	-2.77392	2.90583	1.05486
H	-3.44655	1.50198	2.15137	H	-3.83777	1.57035	1.48293
H	-3.68833	1.94438	0.45882	H	-3.49641	1.92764	-0.21373
C	-2.36710	-0.39146	0.31174	C	-2.11026	-0.33017	0.29285
C	-3.25040	-1.18537	1.27993	C	-3.21788	-1.11607	0.99985
H	-1.45955	-0.97303	0.14680	H	-1.17029	-0.84886	0.47608
H	-4.17277	-0.63447	1.46525	H	-4.17710	-0.62532	0.83542
H	-2.75132	-1.30230	2.24023	H	-3.04742	-1.13104	2.07435
C	-0.85016	3.44866	-1.42166	C	-0.30281	3.67050	-0.64832
H	-0.60593	4.01052	-0.51838	H	-0.27016	4.02170	0.37998

H	-0.31296	3.90456	-2.24994	H	0.47189	4.16789	-1.21766
C	5.04380	-2.35559	0.00304	C	4.66014	-2.18966	0.39601
C	5.41900	-1.09516	0.44747	C	4.94327	-0.84197	0.23023
C	3.76902	-2.55162	-0.50703	C	3.34070	-2.61570	0.33510
C	2.87171	-1.49809	-0.57462	C	2.32088	-1.70651	0.11195
C	3.25275	-0.23943	-0.12752	C	2.59902	-0.35030	-0.05646
C	4.53060	-0.03514	0.38515	C	3.92773	0.07451	0.00579
H	4.81847	0.94512	0.72916	H	4.16698	1.11659	-0.12096
H	6.40794	-0.93342	0.84508	H	5.96378	-0.49546	0.27525
H	3.46875	-3.52679	-0.85474	H	3.10245	-3.66000	0.46158
H	1.87943	-1.63895	-0.96970	H	1.29523	-2.03451	0.06196
N	2.32281	0.79600	-0.21056	N	1.49775	0.49580	-0.27789
O	2.05124	3.08433	0.29182	O	2.33415	2.68314	-0.60764
H	-0.95620	1.69337	-0.60171	H	-0.73216	1.76175	-0.15824
H	-1.91935	3.56446	-1.61512	H	-1.27024	3.90048	-1.08473
H	5.73974	-3.17699	0.05417	H	5.45510	-2.89671	0.56965
C	-3.05702	-0.25403	-1.04512	C	-2.33556	-0.33455	-1.21837
H	-3.97518	0.32381	-0.93891	H	-3.26395	0.18315	-1.45850
H	-2.40745	0.29780	-1.72120	H	-1.52987	0.20846	-1.70747
C	-3.59667	-2.55702	0.70330	C	-3.29317	-2.54587	0.46700
H	-2.68344	-3.14710	0.60816	H	-2.36902	-3.07080	0.71483
H	-4.24508	-3.09587	1.39270	H	-4.09955	-3.08249	0.96455
C	-3.40217	-1.62310	-1.62621	C	-2.41430	-1.76189	-1.75577
H	-2.47989	-2.16845	-1.83385	H	-1.44912	-2.25183	-1.61730
H	-3.91399	-1.50170	-2.57973	H	-2.60207	-1.74062	-2.82808
C	-4.26145	-2.43780	-0.66439	C	-3.49474	-2.56966	-1.04441
H	-5.23154	-1.95073	-0.55012	H	-4.47304	-2.14799	-1.28200
H	-4.45427	-3.42732	-1.07682	H	-3.49871	-3.59626	-1.40801
**Cat.4-IM**				**Cat.4-TS2**			
O	0.54341	2.59031	-0.08608	O	0.46465	2.62406	-0.01808
C	1.63576	1.79487	-0.30361	C	1.53033	1.82642	-0.28599
N	-1.12340	0.15560	1.25026	N	-0.97779	0.00023	1.23932
C	-0.72427	-0.74877	2.35188	C	-0.53391	-1.01021	2.21355
H	-1.55091	-0.89058	3.03586	H	-1.31450	-1.22964	2.93442
H	0.10656	-0.29243	2.87602	H	0.33115	-0.62110	2.73983
H	-0.41490	-1.69927	1.93754	H	-0.25438	-1.91811	1.69326
C	-1.59689	1.44175	1.80674	C	-1.41448	1.20812	1.95696
H	-0.79003	1.86923	2.38761	H	-0.56721	1.59611	2.51056
H	-2.45370	1.26715	2.44491	H	-2.21454	0.97252	2.65161
H	-1.85184	2.11905	1.00648	H	-1.74179	1.96223	1.25695
C	-2.06097	-0.50402	0.27358	C	-1.97201	-0.54641	0.26826
C	-3.42325	-0.83620	0.86806	C	-3.30994	-0.93167	0.89473
H	-1.55613	-1.43321	0.02113	H	-1.50284	-1.45185	-0.11149
H	-3.93973	0.08257	1.14139	H	-3.80001	-0.04167	1.28763
H	-3.31739	-1.43217	1.77066	H	-3.16267	-1.61390	1.72835
C	0.71134	3.96318	-0.36097	C	0.62760	3.99590	-0.31659
H	1.49723	4.39926	0.24882	H	1.44619	4.42903	0.24972
H	0.94709	4.13454	-1.40729	H	0.81126	4.15069	-1.37526
C	4.03474	-2.69304	-0.22783	C	3.99245	-2.60658	-0.36175
C	4.52523	-1.39641	-0.17949	C	4.44890	-1.33314	-0.05142
C	2.65828	-2.87875	-0.21861	C	2.63242	-2.79796	-0.55700

C	1.80224	-1.79393	-0.16293	C	1.75027	-1.73557	-0.44789
C	2.27832	-0.47208	-0.11326	C	2.19588	-0.44702	-0.13175
C	3.67441	-0.30272	-0.12424	C	3.57116	-0.26740	0.06639
H	4.07869	0.69107	-0.09541	H	3.94415	0.71133	0.30537
H	5.59171	-1.22670	-0.18346	H	5.50300	-1.16410	0.10767
H	2.24667	-3.87592	-0.25859	H	2.25305	-3.77755	-0.80384
H	0.73504	-1.95237	-0.16430	H	0.69710	-1.89301	-0.61847
N	1.33100	0.53371	-0.01283	N	1.24070	0.55836	0.02300
O	2.66935	2.30261	-0.72272	O	2.55073	2.29914	-0.76018
H	-0.22318	0.34321	0.71194	H	0.03560	0.28367	0.61513
H	-0.23523	4.43554	-0.12259	H	-0.30333	4.47420	-0.03529
H	4.70550	-3.53612	-0.27273	H	4.68196	-3.43072	-0.45063
C	-2.19818	0.31935	-1.00205	C	-2.17948	0.39607	-0.91443
H	-2.69381	1.26387	-0.78583	H	-2.64954	1.31872	-0.57737
H	-1.21389	0.55023	-1.40055	H	-1.21938	0.66292	-1.34759
C	-4.26218	-1.59854	-0.15797	C	-4.21534	-1.58363	-0.14989
H	-3.79438	-2.56392	-0.35487	H	-3.77045	-2.52727	-0.46912
H	-5.24399	-1.80554	0.26289	H	-5.17480	-1.82747	0.30253
C	-3.02899	-0.44973	-2.02752	C	-3.07635	-0.26082	-1.96185
H	-2.49461	-1.35488	-2.31840	H	-2.57006	-1.13572	-2.37226
H	-3.14014	0.15252	-2.92689	H	-3.23183	0.42797	-2.79018
C	-4.39628	-0.82535	-1.46584	C	-4.41449	-0.68372	-1.36493
H	-4.97344	0.08340	-1.28853	H	-4.97033	0.20623	-1.06539
H	-4.95219	-1.41477	-2.19298	H	-5.01727	-1.19354	-2.11483

Cat.4-PC

O	0.94348	2.67965	-0.13614
C	1.94078	1.80759	-0.31586
N	-1.24227	0.30215	1.22811
C	-0.94215	-0.45833	2.42862
H	-1.78489	-0.51990	3.12342
H	-0.11516	0.01361	2.95440
H	-0.64313	-1.46849	2.16473
C	-1.62918	1.65684	1.58532
H	-0.80199	2.13497	2.10218
H	-2.50118	1.69023	2.24466
H	-1.84432	2.24175	0.69891
C	-2.17930	-0.39417	0.33323
C	-3.62402	-0.48688	0.83770
H	-1.79568	-1.41248	0.26029
H	-4.04650	0.51497	0.91720
H	-3.65018	-0.92275	1.83473
C	1.22813	4.02909	-0.47909
H	2.04993	4.41318	0.11511
H	1.47114	4.11637	-1.53238
C	3.67097	-3.04094	-0.12941
C	4.35532	-1.83754	-0.19433
C	2.28848	-3.02137	-0.01340
C	1.60454	-1.81939	0.03115
C	2.29368	-0.60772	-0.03119
C	3.68338	-0.62525	-0.14281
H	4.22716	0.29811	-0.19303

H	5.43039	-1.83319	-0.28200
H	1.73435	-3.94519	0.03667
H	0.52992	-1.81317	0.11041
N	1.53697	0.56846	0.05032
O	3.02685	2.12209	-0.74648
H	0.56757	0.48017	0.36618
H	0.32355	4.58174	-0.26107
H	4.20401	-3.97695	-0.16866
C	-2.15015	0.19085	-1.07823
H	-2.52266	1.21487	-1.06488
H	-1.12158	0.23133	-1.43230
C	-4.48501	-1.31526	-0.11407
H	-4.12257	-2.34473	-0.12278
H	-5.51117	-1.34967	0.24919
C	-3.00943	-0.63349	-2.03463
H	-2.57828	-1.63081	-2.13626
H	-2.99310	-0.18406	-3.02641
C	-4.44458	-0.75694	-1.53286
H	-4.91454	0.22813	-1.54351
H	-5.02543	-1.38864	-2.20367

Cat.5				Cat.5-RC1			
C	-0.73478	-1.19048	0.18670	O	-2.10929	1.25630	-0.90463
H	-0.82863	-1.25941	1.27898	N	-0.63449	-0.99379	0.04943
H	-1.21713	-2.06726	-0.23943	C	0.34897	-1.30848	-0.97859
C	-0.73478	1.19048	0.18670	H	0.89556	-2.22343	-0.72305
H	-1.21713	2.06726	-0.23943	H	-0.17214	-1.48291	-1.91648
H	-0.82863	1.25941	1.27898	C	0.03331	-0.69673	1.30955
C	0.73478	1.19048	-0.18670	H	0.56248	-1.58090	1.68312
H	1.21713	2.06726	0.23943	H	-0.71790	-0.42281	2.04583
H	0.82863	1.25941	-1.27898	C	-2.73738	1.85908	0.19118
C	0.73478	-1.19048	-0.18670	H	-2.02659	2.23981	0.92896
H	1.21713	-2.06726	0.23943	H	-3.31775	2.70349	-0.17339
H	0.82863	-1.25941	-1.27898	H	-1.59430	0.49652	-0.57440
C	-2.80349	0.00000	-0.01784	H	-3.42413	1.18246	0.70634
H	-3.27403	0.88094	-0.44809	C	1.01739	0.44325	1.13590
H	-3.27403	-0.88094	-0.44809	H	1.53437	0.61794	2.07647
H	-3.00683	-0.00000	1.06071	H	0.46733	1.36026	0.88554
C	2.80349	0.00000	0.01784	C	-1.60880	-2.05628	0.19878
H	3.27403	-0.88094	0.44809	H	-2.34652	-1.77468	0.94535
H	3.00683	-0.00000	-1.06071	H	-2.12063	-2.21867	-0.74596
H	3.27403	0.88094	0.44809	H	-1.14952	-3.00084	0.50868
N	1.39117	0.00000	0.32225	C	1.32912	-0.16399	-1.14413
N	-1.39117	0.00000	-0.32225	H	0.79302	0.71743	-1.51966
				H	2.07620	-0.43518	-1.88620
				N	1.99621	0.12276	0.11295
				C	2.97541	1.17534	-0.03400
				H	3.48529	1.33971	0.91224
				H	3.71765	0.88761	-0.77471
				H	2.53181	2.12786	-0.35049
Cat.5-RC2				Cat.5-TS1			

O	1.27029	2.18189	0.72025	O	-0.56093	1.91690	-0.60492
C	-1.29636	1.84775	-0.91619	C	1.08381	1.64586	0.16474
N	2.78945	-0.20784	1.08311	N	-1.93370	-0.31843	-1.23711
C	1.91444	-1.36718	1.19415	C	-1.50546	-1.49687	-0.48564
H	2.47759	-2.24405	1.53367	H	-1.93252	-2.40082	-0.93024
H	1.14919	-1.15592	1.93684	H	-0.42417	-1.56769	-0.54410
C	3.79057	-0.42991	0.04842	C	-3.38185	-0.15223	-1.12330
H	4.45335	-1.25777	0.32524	H	-3.89269	-0.99074	-1.60608
H	4.39878	0.46607	-0.04548	H	-3.66798	0.75762	-1.64384
C	2.06909	3.08563	0.00988	C	-1.37930	2.90418	-0.00171
H	2.27821	2.75378	-1.00931	H	-1.70662	2.60354	0.98966
H	1.53628	4.03069	-0.05368	H	-0.79704	3.81314	0.07541
C	-5.14930	-1.18993	0.65560	C	5.12992	-1.28041	0.34061
C	-5.11963	-0.34976	-0.44899	C	4.80509	-0.12809	1.04100
C	-4.04010	-1.27266	1.48419	C	4.22680	-1.78683	-0.58354
C	-2.90637	-0.52343	1.21411	C	3.01740	-1.14974	-0.80260
C	-2.88246	0.31499	0.10690	C	2.68442	0.00907	-0.10125
C	-3.99210	0.40343	-0.72872	C	3.59623	0.51572	0.82725
H	-3.96591	1.05681	-1.58577	H	3.36168	1.40921	1.38034
H	-5.97714	-0.27864	-1.09849	H	5.49675	0.27798	1.76233
H	-4.05394	-1.92187	2.34464	H	4.46347	-2.68133	-1.13782
H	-2.03995	-0.57911	1.85194	H	2.31555	-1.53904	-1.52222
N	-1.72417	1.05292	-0.13288	N	1.43398	0.58292	-0.39051
O	-0.80335	2.62085	-1.63480	O	1.24938	2.55936	0.89456
H	1.75467	1.33768	0.78324	H	-1.06760	1.05699	-0.75539
H	3.02522	3.27495	0.50484	H	-2.24279	3.08575	-0.63449
H	-6.02992	-1.77380	0.86818	H	6.07193	-1.77575	0.51186
C	3.12802	-0.73952	-1.27936	C	-3.80973	-0.07145	0.32875
H	3.89418	-0.94306	-2.02371	H	-4.89426	-0.00670	0.37391
H	2.56536	0.14141	-1.61726	H	-3.40808	0.84532	0.77960
C	3.40238	0.11722	2.35605	C	-1.52971	-0.41125	-2.63181
H	4.02296	1.00273	2.24868	H	-1.83988	0.48558	-3.16058
H	2.62997	0.32625	3.09124	H	-0.44865	-0.49196	-2.69261
H	4.02830	-0.69706	2.73570	H	-1.97417	-1.27836	-3.12670
C	1.26047	-1.67368	-0.13859	C	-1.93642	-1.39389	0.96307
H	0.59372	-0.84555	-0.41229	H	-1.41603	-0.55016	1.43680
H	0.64897	-2.56759	-0.04106	H	-1.63723	-2.29735	1.48875
N	2.26485	-1.90017	-1.16164	N	-3.37691	-1.24851	1.05844
C	1.66267	-2.24427	-2.42952	C	-3.81867	-1.21199	2.43410
H	2.43857	-2.44953	-3.16318	H	-4.90342	-1.14708	2.47142
H	1.05441	-3.13867	-2.31836	H	-3.51445	-2.12322	2.94326
H	1.02269	-1.44619	-2.82691	H	-3.40829	-0.35940	2.98985
**Cat.5-IM**				**Cat.5-TS2**			
O	0.22532	2.67596	-0.08340	O	0.36705	2.69428	-0.11176
C	-0.95558	2.00294	-0.26567	C	-0.81441	2.04143	-0.28806
N	1.73919	0.19950	1.06372	N	1.58702	0.07860	1.12757
C	1.92590	-1.23450	1.40568	C	1.66281	-1.37526	1.37864
H	2.91936	-1.33477	1.83108	H	2.60450	-1.58382	1.88251
H	1.18968	-1.49276	2.15723	H	0.84856	-1.64716	2.04001
C	2.66176	0.58937	-0.03497	C	2.63829	0.47466	0.16712
H	3.67246	0.52442	0.35513	H	3.60657	0.31187	0.63633

H	2.43501	1.61384	-0.29702	H	2.51470	1.52950	-0.04061
C	0.26833	3.99668	-0.57985	C	0.41707	4.02423	-0.59002
H	0.10208	4.02412	-1.65263	H	1.41836	4.37959	-0.37691
H	-0.47140	4.62733	-0.09584	H	0.23441	4.06616	-1.65914
C	-3.90443	-2.12637	0.10766	C	-3.88355	-1.97812	0.04074
C	-3.94815	-1.05088	-0.76745	C	-3.71828	-1.06121	-0.98819
C	-2.85925	-2.19343	1.01950	C	-3.00984	-1.93753	1.11736
C	-1.88811	-1.20871	1.05002	C	-1.99459	-0.99589	1.16345
C	-1.91207	-0.11267	0.17042	C	-1.81390	-0.06781	0.13167
C	-2.97893	-0.05950	-0.74409	C	-2.70037	-0.12192	-0.95188
H	-3.03895	0.76558	-1.42777	H	-2.58908	0.58104	-1.75684
H	-4.75061	-0.97770	-1.48637	H	-4.38615	-1.07871	-1.83590
H	-2.80034	-3.01603	1.71603	H	-3.12084	-2.63648	1.93196
H	-1.08799	-1.26685	1.77104	H	-1.33492	-0.95902	2.01554
N	-0.85943	0.78483	0.25745	N	-0.73242	0.80880	0.21926
O	-1.88200	2.57365	-0.82817	O	-1.75320	2.60641	-0.82527
H	0.73571	0.35413	0.73119	H	0.49986	0.37897	0.68204
H	1.26214	4.36873	-0.35745	H	-0.30854	4.65274	-0.08342
H	-4.66430	-2.89097	0.08144	H	-4.67738	-2.70687	0.00445
C	2.48576	-0.32636	-1.22710	C	2.53134	-0.33329	-1.10901
H	3.20730	-0.03472	-1.98514	H	3.34433	-0.04174	-1.76870
H	1.48475	-0.18532	-1.65284	H	1.59096	-0.09061	-1.61943
C	1.90845	1.06232	2.24797	C	1.69529	0.83889	2.37729
H	1.71563	2.08586	1.95580	H	1.59797	1.89423	2.15282
H	1.19541	0.75548	3.00372	H	0.89379	0.53842	3.04324
H	2.91869	0.95913	2.62668	H	2.65295	0.65016	2.85467
C	1.77859	-2.10712	0.17770	C	1.57931	-2.15576	0.08326
H	0.74361	-2.06802	-0.18183	H	0.58725	-2.02284	-0.36518
H	1.98259	-3.13342	0.47044	H	1.69218	-3.21198	0.31343
N	2.71839	-1.70826	-0.85262	N	2.63336	-1.75333	-0.82914
C	2.64973	-2.58751	-1.99968	C	2.60702	-2.53611	-2.04498
H	3.39095	-2.28714	-2.73563	H	3.42941	-2.23885	-2.69076
H	2.86695	-3.60726	-1.69273	H	2.72497	-3.59000	-1.80600
H	1.66550	-2.57847	-2.48261	H	1.67233	-2.41644	-2.60612

Cat.5-PC

O	-0.21314	2.70721	-0.10065
C	-1.31421	1.96877	-0.28426
N	1.74073	0.21567	1.13589
C	2.19250	-1.14958	1.36783
H	3.17694	-1.15224	1.85063
H	1.49460	-1.64083	2.04107
C	2.63379	0.87821	0.19423
H	3.64081	0.97056	0.61815
H	2.25588	1.87761	0.00373
C	-0.32520	4.08366	-0.44002
H	-0.56796	4.20248	-1.49011
H	-1.08315	4.56873	0.16492
C	-3.69775	-2.58592	0.08445
C	-4.02912	-1.44589	-0.63061
C	-2.50265	-2.61302	0.78872
C	-1.65827	-1.51686	0.78053

C	-1.99039	-0.37052	0.05736
C	-3.18879	-0.34266	-0.65443
H	-3.45826	0.53213	-1.21378
H	-4.95312	-1.40798	-1.18579
H	-2.22504	-3.48814	1.35447
H	-0.73784	-1.53999	1.34046
N	-1.07150	0.68659	0.07392
O	-2.35215	2.42287	-0.70784
H	-0.13759	0.48352	0.43497
H	0.64544	4.51464	-0.23287
H	-4.35806	-3.43780	0.09373
C	2.70767	0.10500	-1.10738
H	3.40804	0.59715	-1.77830
H	1.72208	0.11867	-1.59229
C	1.64449	0.95603	2.37857
H	1.26505	1.95410	2.17957
H	0.95488	0.45403	3.05232
H	2.61179	1.04785	2.88456
C	2.27325	-1.92051	0.06495
H	1.26443	-2.03295	-0.35385
H	2.65696	-2.91874	0.26293
N	3.15963	-1.25392	-0.87129
C	3.28286	-1.99568	-2.10519
H	3.98398	-1.49491	-2.76858
H	3.66292	-2.99356	-1.89949
H	2.32831	-2.09956	-2.63667

Cat.6				Cat.6-RC1			
C	-1.18978	-0.77507	-0.68701	O	-3.05174	-0.73183	0.00168
H	-1.18952	-1.16889	-1.70068	C	-3.73090	0.49047	-0.00021
H	-2.06783	-1.16936	-0.18084	H	-3.52606	1.08600	-0.89409
N	0.00002	1.27718	0.00021	H	-4.79915	0.28679	0.02156
N	-0.00002	-1.27718	0.00021	H	-2.09416	-0.53914	-0.01232
C	1.18978	0.77507	-0.68701	H	-3.49383	1.10630	0.87169
H	1.18952	1.16889	-1.70068	C	1.56010	1.44797	-0.31429
H	2.06783	1.16936	-0.18084	H	1.84010	2.19523	0.42395
C	1.18976	-0.77511	-0.68700	H	1.92095	1.78818	-1.28164
H	2.06779	-1.16942	-0.18079	N	-0.28094	-0.16558	-0.01671
H	1.18952	-1.16895	-1.70066	N	2.24093	0.19712	0.01678
C	-1.18976	0.77511	-0.68700	C	0.25110	-0.48092	1.31297
H	-1.18952	1.16895	-1.70066	H	-0.25099	0.15514	2.03710
H	-2.06779	1.16942	-0.18079	H	-0.00465	-1.51172	1.54329
C	0.00002	0.77481	1.37391	C	1.78386	-0.25921	1.32855
H	-0.87746	1.16915	1.88105	H	2.31002	-1.17860	1.57276
H	0.87755	1.16910	1.88103	H	2.06424	0.48773	2.06698
C	-0.00002	-0.77481	1.37391	C	0.02612	1.23239	-0.33557
H	0.87746	-1.16915	1.88105	H	-0.47431	1.86343	0.39402
H	-0.87755	-1.16910	1.88103	H	-0.39372	1.45667	-1.31250
				C	0.36150	-1.03499	-1.00758
				H	-0.06018	-0.80511	-1.98241
				H	0.10589	-2.06417	-0.76976

				C	1.89395	-0.81104	-0.98410
				H	2.42037	-1.73027	-0.73994
				H	2.25508	-0.47071	-1.95131
**Cat.6-RC2**				**Cat.6-TS1**			
O	-0.17638	-0.15745	1.59462	O	-0.32147	2.16826	-0.57454
C	-1.45718	1.92240	-0.27904	C	1.42922	1.77543	-0.09302
C	-0.04788	0.62539	2.75012	C	-0.85674	3.36980	-0.05164
H	0.22679	1.65672	2.52455	H	-1.09866	3.27395	1.00439
H	-1.00873	0.63854	3.25859	H	-0.11246	4.14618	-0.17552
C	-5.27175	-1.62455	-0.15115	C	4.92500	-1.78435	0.16344
C	-4.03670	-1.71632	0.47580	C	4.96155	-0.44176	0.51001
C	-5.57675	-0.50121	-0.90563	C	3.75191	-2.31797	-0.35151
C	-4.65553	0.52530	-1.03441	C	2.63275	-1.51991	-0.51630
C	-3.42224	0.42441	-0.40213	C	2.66296	-0.16967	-0.16895
C	-3.10476	-0.69813	0.35682	C	3.84532	0.36391	0.34771
H	-2.14142	-0.75570	0.83839	H	3.89271	1.40398	0.62235
H	-3.79383	-2.58730	1.06338	H	5.86606	-0.01247	0.91138
H	-6.53306	-0.42055	-1.39654	H	3.70592	-3.35977	-0.62686
H	-4.88091	1.40280	-1.61729	H	1.72065	-1.93119	-0.91746
N	-2.52392	1.47563	-0.55355	N	1.47696	0.55951	-0.36895
O	-0.45152	2.49163	-0.10286	O	1.88400	2.79517	0.28763
H	0.68736	-0.16572	1.13149	H	-0.94734	1.39403	-0.38656
H	0.69148	0.21913	3.44422	H	-1.74994	3.64041	-0.60768
H	-5.99015	-2.42205	-0.05219	H	5.79698	-2.40490	0.29228
C	4.57783	0.66829	-0.02399	C	-4.43716	-0.39181	0.22420
H	5.40368	0.45956	0.65136	H	-5.16262	-0.43182	-0.58396
H	4.80891	1.58909	-0.55354	H	-4.98395	-0.25746	1.15374
N	2.30557	-0.23838	0.28580	N	-2.06713	0.20763	-0.09501
N	4.49830	-0.41705	-1.00024	N	-3.73995	-1.67505	0.28023
C	2.90294	-1.56018	0.50015	C	-2.01684	-0.73707	-1.21969
H	3.06813	-1.68666	1.56678	H	-2.24408	-0.18632	-2.12812
H	2.18440	-2.31178	0.18427	H	-0.99957	-1.10742	-1.30084
C	4.22721	-1.66961	-0.29604	C	-3.02786	-1.88370	-0.97911
H	4.17743	-2.46961	-1.03041	H	-2.52286	-2.84484	-0.93107
H	5.06471	-1.87949	0.36447	H	-3.75852	-1.93351	-1.78211
C	3.24182	0.79073	0.75029	C	-3.42578	0.76027	0.00484
H	3.38778	0.65854	1.81902	H	-3.63464	1.30717	-0.91024
H	2.77980	1.76178	0.59558	H	-3.44141	1.46772	0.82886
C	2.06028	-0.05036	-1.14814	C	-1.74570	-0.50401	1.14975
H	1.58379	0.91619	-1.28372	H	-1.76382	0.21680	1.96241
H	1.35852	-0.81454	-1.47197	H	-0.73323	-0.88603	1.06532
C	3.39757	-0.14422	-1.92350	C	-2.77062	-1.64201	1.37414
H	3.36635	-0.93988	-2.66361	H	-2.27510	-2.60810	1.42179
H	3.61330	0.78439	-2.44596	H	-3.31165	-1.50133	2.30618
**Cat.6-IM**				**Cat.6-TS2**			
O	0.14149	2.59912	0.20827	O	0.07459	2.51179	-0.18788
C	1.32671	1.92174	0.11972	C	-1.14838	1.91681	-0.13302
C	0.22544	4.00490	0.12622	C	0.07687	3.92583	-0.16186
H	0.82105	4.41612	0.93596	H	-0.45510	4.33629	-1.01434
H	0.65471	4.32449	-0.81888	H	-0.37318	4.30450	0.75037
C	4.07819	-2.35083	-0.09055	C	-4.21958	-2.12015	0.08982

C	4.30913	-1.09472	-0.63267	C	-4.30164	-0.89233	0.73155
C	2.87159	-2.57964	0.55782	C	-3.08153	-2.41195	-0.64800
C	1.92551	-1.57516	0.65777	C	-2.05024	-1.49230	-0.74248
C	2.13905	-0.29771	0.11331	C	-2.11658	-0.25288	-0.09549
C	3.36632	-0.08256	-0.53709	C	-3.27066	0.02984	0.64714
H	3.57104	0.88355	-0.95826	H	-3.35357	0.97571	1.14960
H	5.23940	-0.89434	-1.14308	H	-5.17786	-0.64716	1.31219
H	2.66587	-3.54572	0.99328	H	-2.99588	-3.35751	-1.16086
H	0.99592	-1.76197	1.17286	H	-1.17849	-1.72204	-1.33371
N	1.10167	0.61526	0.22081	N	-1.00906	0.58953	-0.18564
O	2.36856	2.55276	-0.01129	O	-2.15941	2.59674	-0.06711
H	-0.49765	0.14792	0.10758	H	0.26531	0.08346	-0.12960
H	-0.79192	4.37188	0.20402	H	1.11820	4.22241	-0.20519
H	4.81884	-3.13052	-0.16938	H	-5.02580	-2.83247	0.16167
C	-3.77518	0.29624	-0.77708	C	3.46529	-0.16783	1.30831
H	-4.06456	0.23144	-1.82100	H	3.65377	-0.68187	2.24597
H	-4.47715	0.95346	-0.27409	H	4.11987	0.69747	1.26490
N	-1.51029	-0.19544	0.01501	N	1.40677	-0.30993	-0.02446
N	-3.87504	-1.03384	-0.18582	N	3.81163	-1.06262	0.20774
C	-1.51723	-1.43864	-0.80125	C	1.45428	-1.78705	0.05085
H	-1.10254	-1.18546	-1.76981	H	0.82433	-2.09643	0.87744
H	-0.85933	-2.14814	-0.31549	H	1.03106	-2.17609	-0.86790
C	-2.97432	-1.94125	-0.88809	C	2.92602	-2.22191	0.23830
H	-3.06150	-2.92737	-0.44361	H	3.22306	-2.90704	-0.54986
H	-3.28922	-2.01058	-1.92419	H	3.05772	-2.72950	1.18909
C	-2.33889	0.84702	-0.64762	C	1.98530	0.26338	1.21194
H	-1.88156	1.05746	-1.60682	H	1.39462	-0.10454	2.04413
H	-2.28090	1.73613	-0.03510	H	1.86682	1.33807	1.15629
C	-2.04110	-0.47538	1.37450	C	2.18010	0.14858	-1.19941
H	-1.96659	0.44361	1.94343	H	2.11589	1.22939	-1.22460
H	-1.39400	-1.21832	1.82566	H	1.69136	-0.24450	-2.08452
C	-3.49644	-0.96856	1.22192	C	3.63345	-0.35692	-1.05837
H	-3.60808	-1.95626	1.65728	H	3.88392	-1.03645	-1.86732
H	-4.17913	-0.29705	1.73240	H	4.33096	0.47451	-1.09295

Cat.6-PC

O	0.36001	2.62527	0.03270
C	1.52203	1.96363	0.02206
C	0.45247	4.04337	0.04865
H	0.96905	4.38628	0.93834
H	0.96965	4.40621	-0.83274
C	4.03360	-2.54567	-0.00110
C	4.49260	-1.24581	-0.14030
C	2.67045	-2.76211	0.14179
C	1.78668	-1.69815	0.14714
C	2.24837	-0.38803	0.00537
C	3.61824	-0.16870	-0.14032
H	3.99026	0.83113	-0.24912
H	5.54855	-1.05666	-0.25356
H	2.28822	-3.76443	0.25280
H	0.73072	-1.87789	0.26397
N	1.28833	0.63065	0.00692

O	2.59794	2.51753	0.02715
H	0.30528	0.34516	0.01497
H	-0.56867	4.40146	0.05243
H	4.72292	-3.37428	-0.00337
C	-3.94580	0.58377	-0.19536
H	-4.42277	0.86174	-1.13208
H	-4.48748	1.07591	0.60875
N	-1.61343	-0.20536	-0.01588
N	-4.08216	-0.86175	-0.02274
C	-1.88416	-1.13594	-1.11639
H	-1.60431	-0.64832	-2.04659
H	-1.24360	-2.00483	-0.99279
C	-3.38099	-1.53528	-1.11441
H	-3.49968	-2.60831	-0.98560
H	-3.86117	-1.26016	-2.05019
C	-2.45026	0.98702	-0.18884
H	-2.16308	1.46875	-1.11902
H	-2.22532	1.67650	0.61889
C	-1.96615	-0.85108	1.25230
H	-1.74202	-0.15711	2.05826
H	-1.32805	-1.72162	1.37889
C	-3.46403	-1.24610	1.24536
H	-3.58682	-2.31868	1.37409
H	-4.00276	-0.75353	2.05104

Cat.7				Cat.7-RC1			
N	-3.52982	-0.56959	0.32800	O	3.63241	1.08940	1.13697
C	-4.05223	-0.84808	-0.99474	N	2.50914	-0.86989	-0.59599
H	-3.28723	-1.28787	-1.62675	C	3.52566	-1.17208	-1.58868
H	-4.86967	-1.56062	-0.91920	H	3.18054	-1.90176	-2.32895
H	-4.43230	0.05184	-1.49633	H	4.40645	-1.57687	-1.09789
C	-2.43681	0.39073	0.29973	H	3.81173	-0.26374	-2.11140
H	-2.17918	0.63313	1.32844	C	3.85956	0.47417	2.37374
H	-2.75503	1.32326	-0.18192	H	4.26882	1.21859	3.05274
C	-1.18564	-0.16782	-0.36902	H	2.94394	0.08269	2.82369
H	-1.32363	-0.22902	-1.45521	H	3.25730	0.41876	0.53464
H	-1.04836	-1.18244	-0.00411	H	4.57886	-0.34629	2.30886
C	2.43255	0.31343	0.35271	C	1.33879	-0.26243	-1.22005
H	2.52687	1.39542	0.32842	H	1.69856	0.51286	-1.89056
H	2.26439	0.03426	1.40132	H	0.79834	-0.99147	-1.83429
C	-4.58994	-0.10589	1.19851	C	0.37703	0.35517	-0.21242
H	-5.37850	-0.85286	1.24780	H	-0.09012	-0.42960	0.37195
H	-4.20464	0.04810	2.20328	H	0.94107	0.98166	0.48702
H	-5.03922	0.83658	0.85921	C	-2.71488	-0.15985	-0.35063
C	1.21806	-0.10482	-0.46747	H	-2.93233	-0.22993	-1.41334
H	1.42821	0.05715	-1.53159	H	-2.11132	-1.03985	-0.09473
H	1.04553	-1.16839	-0.33787	C	2.17668	-2.06364	0.16384
C	-0.04444	1.93463	-0.59621	H	1.49018	-1.82792	0.96988
H	-0.18190	1.92839	-1.68522	H	1.71868	-2.83554	-0.46439
H	-0.86123	2.49756	-0.15581	H	3.08058	-2.47513	0.60374
H	0.87216	2.47287	-0.37869	C	-0.23462	2.46580	-1.18238

C	4.81652	0.41342	0.45804	H	-0.06143	3.06501	-0.27991
H	4.86085	0.26360	1.54466	H	0.69122	2.44749	-1.74969
H	5.73750	0.02467	0.03031	H	-0.98328	2.97151	-1.78658
H	4.77979	1.48307	0.26753	C	-1.92175	1.12269	-0.13478
C	3.76473	-1.67362	0.02559	H	-2.52980	1.94765	-0.49632
H	3.69980	-1.97088	1.08062	H	-1.74496	1.30646	0.92983
H	2.97337	-2.18151	-0.51657	N	-0.67087	1.11610	-0.88190
H	4.71370	-2.03161	-0.36551	N	-3.97761	-0.16516	0.37217
N	3.67791	-0.23861	-0.15458	C	-4.89899	-1.12734	-0.19578
N	0.01218	0.59440	-0.04194	H	-5.84820	-1.08301	0.33286
				H	-5.08236	-0.89537	-1.24173
				H	-4.52945	-2.15938	-0.13605
				C	-3.80016	-0.42018	1.78756
				H	-4.76236	-0.36912	2.29130
				H	-3.36689	-1.40958	1.98496
				H	-3.15159	0.32551	2.23609
**Cat.7-RC2**				**Cat.7-TS1**			
O	0.81743	1.98530	-1.44130	O	1.24757	2.13013	-0.88534
C	3.64330	1.68901	-0.32226	C	2.87256	1.25629	-0.94065
N	-0.31290	1.54062	1.13569	N	-0.19136	1.76901	1.34665
C	0.78009	1.50100	2.09181	C	0.72270	1.79652	2.48095
H	0.43064	1.33038	3.11578	H	0.19596	1.66644	3.42997
H	1.31919	2.44414	2.06979	H	1.24425	2.74896	2.50474
H	1.46993	0.70474	1.82872	H	1.45584	1.00323	2.37839
C	0.62570	3.33901	-1.74626	C	1.26195	3.51463	-1.18450
H	1.07464	3.53371	-2.71735	H	1.64443	4.09885	-0.35102
H	-0.43184	3.60705	-1.80954	H	1.90368	3.65932	-2.04411
C	5.85817	-2.92437	0.23674	C	5.41727	-2.82660	0.40034
C	6.44193	-1.67224	0.37250	C	5.77117	-1.75134	-0.40121
C	4.51440	-3.02264	-0.09250	C	4.14757	-2.86382	0.95934
C	3.75483	-1.88008	-0.28586	C	3.24629	-1.84065	0.71935
C	4.34461	-0.63027	-0.14781	C	3.59613	-0.75658	-0.08534
C	5.69266	-0.52383	0.18194	C	4.87458	-0.72289	-0.64604
H	6.14192	0.45056	0.28636	H	5.16734	0.10313	-1.27148
H	7.48576	-1.58612	0.62790	H	6.75424	-1.70822	-0.84305
H	4.05254	-3.99065	-0.20038	H	3.85541	-3.69233	1.58505
H	2.71053	-1.94484	-0.54256	H	2.25965	-1.86742	1.15272
N	3.54960	0.49777	-0.34873	N	2.61323	0.23178	-0.27548
O	3.64544	2.85412	-0.31180	O	3.57992	1.94885	-1.58314
H	0.44399	1.82217	-0.55380	H	0.73485	1.95351	-0.02990
H	1.10112	4.00206	-1.02157	H	0.25584	3.84123	-1.43040
H	6.44634	-3.81499	0.38653	H	6.11966	-3.62291	0.58621
C	-1.00112	0.25552	1.08395	C	-0.85763	0.46902	1.25974
H	-0.23924	-0.51307	0.98838	H	-0.08084	-0.28924	1.27476
H	-1.53835	0.05975	2.01901	H	-1.48866	0.30424	2.13836
C	-1.97357	0.14247	-0.08360	C	-1.69893	0.30245	0.00042
H	-2.81621	0.80443	0.08113	H	-2.59114	0.91379	0.07485
H	-1.47855	0.47887	-1.00094	H	-1.13870	0.66283	-0.86900
C	-4.87043	-0.99279	0.32494	C	-4.54351	-1.00541	0.16361
H	-4.75584	-1.75924	1.08717	H	-4.45685	-1.76572	0.93556
H	-4.67754	-0.03042	0.81567	H	-4.44597	-0.03494	0.66628

C	-1.21212	2.64062	1.44379	C	-1.14124	2.87162	1.43875
H	-1.97138	2.74352	0.67594	H	-1.74879	2.92787	0.54223
H	-1.71322	2.50318	2.40835	H	-1.80318	2.76227	2.30229
H	-0.64773	3.56792	1.48269	H	-0.59769	3.80603	1.53874
C	-1.57348	-2.04007	-1.00350	C	-1.08370	-1.84607	-0.87310
H	-1.51504	-1.72106	-2.05141	H	-0.97647	-1.53734	-1.92016
H	-0.56987	-2.00127	-0.58974	H	-0.11822	-1.71815	-0.39260
H	-1.90083	-3.07630	-0.98224	H	-1.32930	-2.90476	-0.85476
C	-3.83010	-1.26862	-0.75315	C	-3.39881	-1.22368	-0.81738
H	-4.01003	-2.27084	-1.13282	H	-3.48328	-2.23741	-1.19963
H	-3.94439	-0.58624	-1.60137	H	-3.48152	-0.55514	-1.68026
N	-2.47547	-1.21987	-0.21890	N	-2.10072	-1.08815	-0.17022
N	-6.23338	-1.05607	-0.18061	N	-5.84964	-1.14018	-0.46293
C	-7.17868	-1.26194	0.89723	C	-6.87626	-1.41305	0.52140
H	-8.18460	-1.34468	0.49271	H	-7.83372	-1.54965	0.02437
H	-6.94838	-2.18430	1.42420	H	-6.64147	-2.32651	1.06169
H	-7.17690	-0.44305	1.62832	H	-6.99128	-0.60420	1.25476
C	-6.59350	0.12427	-0.94024	C	-6.20671	0.02596	-1.24541
H	-7.60213	0.01336	-1.33064	H	-7.16385	-0.14059	-1.73355
H	-6.56373	1.03912	-0.33382	H	-6.29275	0.93273	-0.63228
H	-5.92469	0.25931	-1.78415	H	-5.46856	0.21276	-2.01872
Cat.7-IM				Cat.7-TS2			
O	1.08422	2.49500	-0.08534	O	0.93561	2.44849	0.03097
C	2.21938	1.85729	-0.50799	C	2.08581	1.87149	-0.40685
N	0.33699	0.06198	1.91513	N	0.43960	-0.23163	1.78857
C	1.12222	-0.58263	2.98900	C	1.23072	-1.03930	2.72943
H	0.46345	-0.83410	3.81231	H	0.59805	-1.40384	3.53433
H	1.88422	0.11089	3.32272	H	2.02140	-0.42301	3.14244
H	1.58798	-1.47773	2.59868	H	1.66919	-1.87922	2.20523
C	0.91633	3.82184	-0.53386	C	0.69871	3.77017	-0.41097
H	0.85447	3.87154	-1.61697	H	0.62246	3.81722	-1.49277
H	-0.01591	4.16935	-0.10266	H	-0.24375	4.06824	0.03332
C	5.35608	-2.15180	-0.64660	C	5.47145	-1.89613	-0.67013
C	5.57929	-0.80844	-0.91012	C	5.66082	-0.52318	-0.59681
C	4.10051	-2.53503	-0.19303	C	4.18827	-2.40096	-0.51758
C	3.10168	-1.59607	-0.01043	C	3.11879	-1.54800	-0.30032
C	3.30698	-0.22971	-0.27066	C	3.29530	-0.16165	-0.21940
C	4.58367	0.14019	-0.73023	C	4.59643	0.33542	-0.37270
H	4.77986	1.17235	-0.94858	H	4.76326	1.39543	-0.32190
H	6.54786	-0.48561	-1.26208	H	6.65191	-0.11088	-0.70987
H	3.89477	-3.57358	0.01771	H	4.01385	-3.46431	-0.57496
H	2.12913	-1.90983	0.33468	H	2.12320	-1.95022	-0.19946
N	2.24648	0.62368	-0.01243	N	2.17898	0.62484	0.06498
O	2.99727	2.45172	-1.24463	O	2.84191	2.48773	-1.14049
H	1.03429	0.30931	1.14205	H	1.22020	0.17948	0.95473
H	1.72930	4.46093	-0.20176	H	1.48524	4.44293	-0.08368
H	6.13732	-2.88071	-0.79162	H	6.30527	-2.55725	-0.84374
C	-0.65247	-0.89017	1.34373	C	-0.57473	-1.07307	1.11878
H	-0.09356	-1.77352	1.05823	H	-0.04118	-1.90782	0.67785
H	-1.34430	-1.16348	2.13404	H	-1.25188	-1.47133	1.87184
C	-1.40221	-0.32687	0.14460	C	-1.36648	-0.33897	0.04525

H	-2.09115	0.44054	0.47675	H	-2.03437	0.37476	0.51301
H	-0.70071	0.14788	-0.54791	H	-0.69096	0.23071	-0.59869
C	-4.49907	-0.72823	-0.07719	C	-4.47250	-0.72914	-0.07329
H	-4.68464	-1.70168	0.36946	H	-4.62909	-1.76275	0.22436
H	-4.17142	-0.06705	0.73506	H	-4.10293	-0.19880	0.81347
C	-0.28632	1.30480	2.41778	C	-0.15135	0.91284	2.49874
H	-0.75932	1.83172	1.60354	H	-0.65695	1.56316	1.80064
H	-1.01314	1.04693	3.18019	H	-0.84939	0.55805	3.25299
H	0.48951	1.92966	2.84103	H	0.64465	1.47141	2.97736
C	-1.34594	-2.10953	-1.46287	C	-1.39982	-1.84609	-1.82441
H	-1.06082	-1.48997	-2.32120	H	-1.15756	-1.09771	-2.58828
H	-0.43327	-2.46677	-0.99491	H	-0.46526	-2.26807	-1.46619
H	-1.89047	-2.97433	-1.83205	H	-1.96447	-2.64483	-2.29785
C	-3.39321	-0.90637	-1.10952	C	-3.42452	-0.73006	-1.17853
H	-3.72930	-1.65025	-1.82635	H	-3.79896	-1.35414	-1.98529
H	-3.21954	0.01628	-1.67144	H	-3.28405	0.27194	-1.59568
N	-2.15944	-1.38945	-0.50124	N	-2.15989	-1.29120	-0.72078
N	-5.74178	-0.25466	-0.66630	N	-5.74809	-0.18517	-0.51303
C	-6.86865	-0.53404	0.19946	C	-6.82570	-0.61903	0.35176
H	-7.78884	-0.20844	-0.27964	H	-7.77230	-0.23408	-0.01990
H	-6.94194	-1.60277	0.38317	H	-6.88178	-1.70445	0.36184
H	-6.79753	-0.02601	1.17006	H	-6.70685	-0.27489	1.38741
C	-5.69671	1.15828	-0.98490	C	-5.72924	1.26136	-0.59832
H	-6.62660	1.45406	-1.46427	H	-6.68483	1.61723	-0.97547
H	-5.56060	1.78541	-0.09400	H	-5.55123	1.73680	0.37527
H	-4.88612	1.37349	-1.67356	H	-4.95692	1.59844	-1.28219

Cat.7-PC

O	-3.15438	-2.36622	-0.05925
C	-3.78789	-1.20583	-0.26085
N	-0.24082	-0.87592	1.11236
C	0.08251	0.10981	2.12706
H	0.98520	-0.13410	2.69468
H	-0.74214	0.18250	2.83254
H	0.22292	1.08332	1.66638
C	-3.90531	-3.54003	-0.33898
H	-4.79053	-3.58936	0.28546
H	-4.19667	-3.57210	-1.38303
C	-3.71676	3.93974	-0.28801
C	-4.78055	3.05248	-0.23836
C	-2.42313	3.44144	-0.23217
C	-2.19928	2.07933	-0.13413
C	-3.26981	1.18666	-0.08001
C	-4.57056	1.68587	-0.13020
H	-5.40380	1.01100	-0.09062
H	-5.79302	3.42233	-0.27778
H	-1.58019	4.11295	-0.27197
H	-1.19175	1.69795	-0.10676
N	-2.96876	-0.17540	0.05245
O	-4.92535	-1.13389	-0.66711
H	-2.02341	-0.41810	0.36211
H	-3.24650	-4.36832	-0.11312

H	-3.89178	5.00021	-0.36938
C	0.65622	-0.85338	-0.03708
H	0.24659	-1.53915	-0.77488
H	0.61625	0.14355	-0.47160
C	2.12310	-1.19135	0.23222
H	2.51970	-0.47678	0.94595
H	2.21543	-2.18130	0.69258
C	4.55924	0.66177	-0.48410
H	4.28458	1.17813	-1.40039
H	3.89951	1.04726	0.30308
C	-0.40707	-2.19400	1.69739
H	-0.64692	-2.91307	0.92104
H	0.48124	-2.54067	2.23275
H	-1.23357	-2.17156	2.40345
C	2.78432	-2.31843	-1.78430
H	3.19365	-3.20449	-1.28292
H	1.73931	-2.51469	-2.00362
H	3.30337	-2.19558	-2.73124
C	4.32076	-0.82551	-0.71206
H	4.90752	-1.12152	-1.57753
H	4.68571	-1.41996	0.13174
N	2.91875	-1.11372	-0.98813
N	5.95224	0.96462	-0.19207
C	6.25080	2.35660	-0.45777
H	7.30461	2.54844	-0.27040
H	6.04398	2.59009	-1.49891
H	5.66794	3.04543	0.16754
C	6.31267	0.62941	1.17083
H	7.37094	0.82334	1.32749
H	5.74832	1.21345	1.90971
H	6.13593	-0.42285	1.36950

Cat.8				Cat.8-RC1			
C	0.00000	0.48668	-0.09922	O	2.27539	1.50446	-0.77004
H	0.00000	1.02211	-1.04720	N	1.55746	-0.96803	0.39421
H	0.00000	1.24115	0.69039	C	2.56747	-2.00139	0.24539
C	-1.26549	-0.34623	0.01019	H	2.25720	-2.96655	0.65497
H	-1.25191	-0.91584	0.94829	H	3.48016	-1.69282	0.74709
H	-1.29078	-1.08068	-0.79507	H	2.79141	-2.14074	-0.80948
C	-3.67845	-0.27552	0.03357	C	2.48942	2.38087	0.29901
H	-4.53117	0.39824	0.01029	H	2.68852	3.37065	-0.10537
H	-3.77701	-0.96752	-0.80076	H	1.62156	2.46037	0.95867
H	-3.73286	-0.86217	0.95761	H	2.06183	0.62843	-0.39066
C	1.26549	-0.34623	0.01019	H	3.34835	2.09417	0.91210
H	1.29078	-1.08068	-0.79507	C	0.27277	-1.33995	-0.18383
H	1.25191	-0.91584	0.94829	H	0.42973	-1.52612	-1.24568
N	2.45189	0.48364	-0.10455	H	-0.10271	-2.27393	0.24774
H	2.42260	1.18128	0.62497	C	-0.74922	-0.23098	-0.00090
N	-2.45189	0.48364	-0.10455	H	-0.86460	-0.03032	1.06450
H	-2.42260	1.18128	0.62497	H	-0.37029	0.68097	-0.45861
C	3.67845	-0.27552	0.03357	C	-2.09662	-0.58132	-0.61471

H	4.53117	0.39824	0.01029	H	-1.95684	-0.78699	-1.67441
H	3.73286	-0.86217	0.95761	H	-2.47288	-1.50576	-0.15832
H	3.77701	-0.96752	-0.80076	H	1.42497	-0.78271	1.37861
				N	-3.05851	0.50520	-0.50304
				H	-3.81114	0.32368	-1.14888
				C	-3.62923	0.64750	0.82304
				H	-4.42947	1.38238	0.79305
				H	-4.03864	-0.28651	1.22480
				H	-2.88058	1.00760	1.52401
**Cat.8-RC2**				**Cat.8-TS1**			
O	-0.83847	1.77177	-1.20029	O	-0.36974	2.01166	-0.55387
C	2.03896	1.88908	-0.18826	C	1.42568	1.52730	-0.71007
N	-1.60110	0.90912	1.38223	N	-1.60273	1.02957	1.59560
C	-0.52000	0.93326	2.35264	C	-0.74180	1.09787	2.76846
H	-0.82310	0.57447	3.34008	H	-1.22397	0.70176	3.66400
H	-0.14622	1.94802	2.45556	H	-0.46232	2.13088	2.95245
H	0.29504	0.30532	2.00244	H	0.16291	0.52586	2.58479
C	-1.33056	3.07614	-1.32616	C	-0.65473	3.39632	-0.59853
H	-1.07106	3.44387	-2.31616	H	-0.34118	3.89934	0.31381
H	-2.41843	3.12300	-1.22899	H	-0.12059	3.82067	-1.43925
C	5.02611	-2.30644	-0.12794	C	4.76773	-2.11410	0.01219
C	5.39256	-0.99244	0.12915	C	4.89695	-0.85569	-0.55682
C	3.71002	-2.60072	-0.45201	C	3.52733	-2.51810	0.48582
C	2.76270	-1.59190	-0.51965	C	2.43328	-1.67497	0.39186
C	3.13544	-0.27929	-0.26039	C	2.55719	-0.40864	-0.17847
C	4.45455	0.02376	0.06456	C	3.80642	-0.00565	-0.65419
H	4.73490	1.04546	0.26356	H	3.92566	0.96777	-1.09892
H	6.41330	-0.75435	0.38142	H	5.85443	-0.52771	-0.92986
H	3.41637	-3.61813	-0.65359	H	3.40889	-3.49364	0.93031
H	1.73777	-1.80887	-0.77055	H	1.46788	-1.98607	0.75686
N	2.15477	0.70899	-0.33656	N	1.39083	0.37565	-0.23285
O	1.83715	3.02930	-0.05824	O	1.95331	2.45787	-1.20589
H	-1.06504	1.45936	-0.30121	H	-0.80166	1.59742	0.26468
H	-0.89708	3.75925	-0.59312	H	-1.72199	3.54116	-0.74372
H	5.76078	-3.09332	-0.07587	H	5.62011	-2.77005	0.08451
C	-2.19797	-0.41129	1.22939	C	-2.08951	-0.32080	1.32421
H	-1.41886	-1.08726	0.87865	H	-1.22231	-0.94828	1.12753
H	-2.54721	-0.80395	2.19036	H	-2.59238	-0.73406	2.20279
C	-3.34267	-0.38109	0.23118	C	-3.02554	-0.33777	0.12839
H	-4.09783	0.32215	0.58288	H	-3.85963	0.33670	0.32214
H	-2.97580	-0.00494	-0.72208	H	-2.50374	0.04302	-0.74788
C	-3.96417	-1.75455	0.02541	C	-3.54364	-1.73737	-0.16976
H	-3.19475	-2.43832	-0.32829	H	-2.69485	-2.39487	-0.34767
H	-4.31420	-2.14188	0.99070	H	-4.06664	-2.12441	0.71385
H	-2.31967	1.55310	1.68204	H	-2.39991	1.63346	1.74438
N	-5.02812	-1.74086	-0.96771	N	-4.38549	-1.77698	-1.35582
H	-5.19621	-2.69142	-1.25862	H	-4.46292	-2.73752	-1.65202
C	-6.27882	-1.19279	-0.47804	C	-5.72481	-1.26264	-1.14063
H	-7.05175	-1.33303	-1.22923	H	-6.32726	-1.44720	-2.02619
H	-6.62225	-1.65395	0.45512	H	-6.23339	-1.71480	-0.28144
H	-6.18389	-0.12439	-0.30185	H	-5.69928	-0.18786	-0.98028

Cat.8-IM				Cat.8-TS2			
O	0.19855	2.46830	-0.38408	O	0.12469	2.47634	-0.34114
C	1.25931	1.64110	-0.64783	C	1.17364	1.66506	-0.65092
C	0.17789	3.69119	-1.08888	C	0.07599	3.71385	-1.02358
H	0.13059	3.53028	-2.16181	H	-0.00710	3.56928	-2.09621
H	-0.71498	4.21141	-0.76096	H	-0.80717	4.22217	-0.65506
C	3.83392	-2.70885	0.08902	C	3.83233	-2.65655	-0.00690
C	4.12880	-1.58690	-0.67014	C	4.15620	-1.46646	-0.63973
C	2.65218	-2.71820	0.81985	C	2.59809	-2.75673	0.62108
C	1.79595	-1.63396	0.78691	C	1.71540	-1.69223	0.61375
C	2.07561	-0.48662	0.02202	C	2.02920	-0.47957	-0.02075
C	3.27629	-0.49302	-0.71063	C	3.27978	-0.39148	-0.65165
H	3.52591	0.36266	-1.30754	H	3.55082	0.51835	-1.15088
H	5.04154	-1.55529	-1.24646	H	5.11014	-1.36428	-1.13472
H	2.39405	-3.57724	1.42048	H	2.31642	-3.67153	1.11953
H	0.88104	-1.66036	1.35838	H	0.75839	-1.79825	1.09908
N	1.14237	0.53337	0.07737	N	1.08296	0.53477	0.05479
O	2.10753	2.00022	-1.45468	O	2.00239	2.02479	-1.47149
H	1.05245	4.29453	-0.86454	H	0.95616	4.31406	-0.81569
H	4.50420	-3.55316	0.11171	H	4.52129	-3.48585	-0.00417
N	-1.00801	0.62647	1.76634	N	-0.85410	0.47578	1.77173
H	-1.02889	-0.23528	2.30359	H	-0.83562	-0.42923	2.22888
H	-0.17601	0.54208	1.08761	H	0.02830	0.46739	0.95317
C	-0.74916	1.75212	2.68129	C	-0.54221	1.51017	2.76421
H	0.19163	1.57475	3.18809	H	0.42988	1.29922	3.19568
H	-0.68436	2.65993	2.09671	H	-0.51114	2.47097	2.26535
H	-1.55228	1.82521	3.40481	H	-1.29211	1.52791	3.54899
C	-2.27202	0.74630	1.00399	C	-2.16810	0.65219	1.12801
H	-2.17922	1.63572	0.39145	H	-2.13620	1.60145	0.60411
H	-3.07524	0.89652	1.71908	H	-2.93001	0.71481	1.90221
C	-2.51250	-0.48744	0.15591	C	-2.46435	-0.48549	0.16848
H	-2.53411	-1.36491	0.80085	H	-2.43090	-1.42836	0.71314
H	-1.69115	-0.61918	-0.54522	H	-1.69561	-0.52916	-0.60028
C	-3.81734	-0.37325	-0.62099	C	-3.82240	-0.31292	-0.49792
H	-3.77086	0.50909	-1.25574	H	-3.83199	0.63374	-1.03430
H	-4.64350	-0.21733	0.08375	H	-4.59827	-0.24989	0.27525
N	-4.04609	-1.52362	-1.47986	N	-4.09997	-1.36716	-1.46021
H	-4.73642	-1.26710	-2.16817	H	-4.84590	-1.05353	-2.06140
C	-4.52588	-2.69674	-0.77362	C	-4.50516	-2.62214	-0.85552
H	-5.40148	-2.49962	-0.14489	H	-5.33185	-2.51732	-0.14375
H	-4.79204	-3.46549	-1.49415	H	-4.81539	-3.31252	-1.63541
H	-3.74565	-3.10328	-0.13527	H	-3.67147	-3.07836	-0.32800

Cat.8-PC			
O	0.33064	2.54849	-0.37089
C	1.34879	1.72994	-0.66638
C	0.31772	3.79345	-1.05669
H	0.24627	3.64245	-2.12819
H	-0.55949	4.31514	-0.69706
C	3.70426	-2.80439	0.09257
C	4.04127	-1.72733	-0.71108
C	2.55405	-2.72911	0.86581

C	1.75817	-1.59877	0.83302
C	2.09737	-0.51310	0.02196
C	3.25346	-0.58600	-0.75476
H	3.52781	0.23914	-1.38259
H	4.93171	-1.76669	-1.31872
H	2.27082	-3.55454	1.49952
H	0.86562	-1.54909	1.43541
N	1.23705	0.58904	0.05019
O	2.21444	2.01474	-1.46280
H	1.20941	4.36771	-0.83072
H	4.32441	-3.68562	0.11722
N	-1.08481	0.50262	1.87350
H	-1.15789	-0.36967	2.37772
H	0.42676	0.52710	0.67719
C	-0.84071	1.57211	2.82578
H	0.03960	1.33729	3.41777
H	-0.65033	2.49698	2.28794
H	-1.67961	1.74036	3.50698
C	-2.30739	0.69681	1.10598
H	-2.19598	1.61742	0.53600
H	-3.17204	0.83300	1.76493
C	-2.55647	-0.46750	0.16285
H	-2.59444	-1.38990	0.74265
H	-1.72127	-0.56131	-0.52967
C	-3.84370	-0.29817	-0.63131
H	-3.78843	0.63084	-1.19579
H	-4.68633	-0.19771	0.06449
N	-4.05703	-1.37756	-1.58397
H	-4.75111	-1.07399	-2.24921
C	-4.52457	-2.60961	-0.97730
H	-5.40134	-2.47444	-0.33358
H	-4.78480	-3.31885	-1.75869
H	-3.73994	-3.06010	-0.37493
